# Non-Secreted Clusterin Isoforms Are Translated in Rare Amounts from Distinct Human mRNA Variants and Do Not Affect Bax-Mediated Apoptosis or the NF-κB Signaling Pathway

**DOI:** 10.1371/journal.pone.0075303

**Published:** 2013-09-20

**Authors:** Hans Prochnow, Rene Gollan, Philipp Rohne, Matthias Hassemer, Claudia Koch-Brandt, Markus Baiersdörfer

**Affiliations:** 1 Institute of Pharmacy and Biochemistry, Therapeutical Life Sciences, Johannes Gutenberg - University, Mainz, Mainz, Germany; 2 Department of Neurology, University Medical Center Mainz, Johannes Gutenberg-University, Mainz, Mainz, Germany; Complutense University, Spain

## Abstract

Clusterin, also known as apolipoprotein J, is expressed from a variety of tissues and implicated in pathological disorders such as neurodegenerative diseases, ischemia and cancer. In contrast to secretory clusterin (sCLU), which acts as an extracellular chaperone, the synthesis, subcellular localization and function(s) of intracellular CLU isoforms is currently a matter of intense discussion. By investigating human CLU mRNAs we here unravel mechanisms leading to the synthesis of distinct CLU protein isoforms and analyze their subcellular localization and their impact on apoptosis and on NF-κB-activity. Quantitative PCR-analyses revealed the expression of four different stress-inducible CLU mRNA variants in non-cancer and cancer cell lines. In all cell lines variant 1 represents the most abundant mRNA, whereas all other variants collectively account for no more than 0.34% of total CLU mRNA, even under stressed conditions. Overexpression of CLU cDNAs combined with *in vitro* mutagenesis revealed distinct translational start sites including a so far uncharacterized non-canonical CUG start codon. We show that all exon 2-containing mRNAs encode sCLU and at least three non-glycosylated intracellular isoforms, CLU_1‑449_, CLU_21‑449_ and CLU_34‑449_, which all reside in the cytosol of unstressed and stressed HEK‑293 cells. The latter is the only form expressed from an alternatively spliced mRNA variant lacking exon 2. Functional analysis revealed that none of these cytosolic CLU forms modulate caspase-mediated intrinsic apoptosis or significantly affects TNF-α-induced NF-κB-activity. Therefore our data challenge some of the current ideas regarding the physiological functions of CLU isoforms in pathologies.

## Introduction

Clusterin (CLU), also known as Apolipoprotein J, is a secreted glycoprotein constitutively expressed from a broad spectrum of tissues, especially in brain, neuronal tissue, liver, adrenal glands and testis. It is found in body fluids including serum, cerebrospinal fluid, mother’s milk, semen and urine. CLU has been identified and characterized by virtue of its upregulation in degenerative conditions. Thus its expression has been shown to be induced in a broad range of pathologies such as Alzheimer’s disease [1,2], spongiform encephalopathies [3], ischemic injury of the hippocampus and the heart [4,5], myocarditis [6], atherosclerosis [7,8] as well as cancer [9]. Common to these diverse pathological disorders is the induction of a cellular stress response due to injury, increased oxidative or proteotoxic stress or dysregulation of particular signal transduction pathways [10]. This in turn poses a severe threat for cells but can be antagonized by upregulation of a “defense machinery” including proteolytic, metabolic and DNA/RNA modifying enzymes, detoxifying proteins and molecular chaperones altogether known as heat shock proteins [11].

As part of this cellular stress program, CLU mRNA expression is induced. This transcriptional response is mediated by different elements in the CLU promoter region like AP‑1 elements [12,13], TCF-binding sites [14], putative binding sites for NF-κB and Stat1 [15] and a CLE (Clusterin element), a sequence with high similarity to HSEs (heat shock elements) [16,17,18] resulting in a concomitant increase in CLU protein synthesis and secretion. Secreted CLU (sCLU) has been shown to bind a whole set of target proteins via interaction with hydrophobic domains and exhibits properties similar to small heat shock proteins (sHsps) i.e. binding to unfolding client proteins, preventing their aggregation as well as initiating their disposal by uptake into non-professional phagocytic cells, thereby exerting a cytoprotective function in the affected tissue [19,20,21,22]. This is exemplified by various studies reporting increased proliferation rates, cell-viability and invasiveness of cells upon CLU overexpression under stress conditions [23,24,25,26]. In accordance, siRNA- or antisense oligodeoxynucleotide-mediated CLU knockdown results in opposite effects i.e. in a decrease in cell proliferation and viability as well as in an increase in the sensitivity of cells to chemotherapeutic drugs [16,27,28,29]. However, in some studies sCLU has also been reported to exert anti-proliferative activity leading to an arrest in the G_0_-phase of the cell cycle or to even induce apoptosis [26,30,31].

Apart from sCLU non-secreted, intracellular forms of CLU have been found within stressed cells. The functions, properties, subcellular localization and the biogenesis of these CLU proteins are not clear, yet. Several mechanisms as to their biogenesis are being discussed: 1) Retrotranslocation of a post-translationally modified sCLU precursor form from the endoplasmic reticulum (ER) to the cytosol after stress induction presumably by the ER-associated protein degradation pathway [32,33]. 2) Internal translation initiation at AUG codons downstream the ER signal sequence coding region (SSCR) would generate non-secreted CLU isoforms [34,35]. 3) Translational initiation at AUG codons upstream of the SSCR on exon 1 of individual CLU mRNA variants could result in the synthesis of N‑terminally elongated CLU proteins with presumably impaired functionality of the ER signal sequence [36]. 4) Alternative splicing of CLU mRNA could generate an mRNA lacking exon 2 which includes the SSCR. Translation of this mRNA would initiate at an AUG on exon 3 leading to synthesis of an N-terminal truncated, non-secreted CLU isoform. Such a splicing event has been observed in the human mammary gland carcinoma cell line MCF‑7 subjected to ionizing radiation. Owing to the presence of a putative nuclear localization sequence this CLU isoform - termed nuclear Clusterin (nCLU) - has been suggested to translocate into the nucleus of cells where it might act as a pro-death factor [37,38,39]. Functions for these intracellular CLU isoforms are still debated; both, the activation and inhibition of the intrinsic apoptotic pathway [40,41,42,43] as well as the NF-κB signaling cascade have been reported [44,45].

Despite the controversial data on the function of different CLU isoforms, modulating their expression is currently considered an attractive strategy in cancer treatment. Thus, therapies combining conventional chemotherapeutic drugs with an antisense oligonucleotide strategy targeting CLU to block its cytoprotective effect have been developed for the treatment of non-small cell lung cancer and prostate cancer, of which the latter is currently in phase III of clinical trials [9,46]. If, however, expression of CLU isoforms with pro-apoptotic functions would be inhibited in cancer cells, this could undermine the ultimate goal of this strategy. To limit such therapeutic risks a stringent analysis of CLU mRNA expression profiles and of the encoded secreted and intracellular proteins is fundamental. Data gathered from such investigations will not only support further studies on CLU-based cancer therapy but will also help to unravel the contradictory data on the protein’s role in pathologies such as brain ischemia [4,47,48], Alzheimer’s disease [49,50], atherosclerosis [7,8,22,51] and cancer [41,42,52,53].

For the first time we here present a quantitative analysis of distinct CLU mRNA variants and a characterization of the encoded CLU isoforms. We use non-malignant HEK‑293 cells as well as prostate cancer (PC‑3), mammary gland carcinoma (MCF‑7) and colorectal adenocarcinoma cells (Caco‑2) since expression of intracellular CLU isoforms and/or different CLU mRNA variants has been reported in these cells and for these types of cancer [14,39,54]. By using the proteasome inhibitor MG‑132, we induced proteotoxic stress leading to the induction of distinct CLU mRNA variants and the concomitant appearance of non-secreted CLU isoforms. *In vitro* mutagenesis and overexpression of individual CLU forms from engineered cDNAs allowed us to characterize the biogenesis, the subcellular location and the impact of distinct isoforms on Bcl-2-associated X protein (Bax)-mediated apoptosis and on NF-κB signaling.

## Materials and Methods

For a detailed description, please refer to Protocol S1.

### Cell culture

HEK-293, PC-3, MCF-7 and Caco-2 cell lines were grown in the presence of 10% FBS at 37 °C in a humidified atmosphere with 5% CO_2_. Proteasome activity was inhibited by incubation of the cells in presence of 10 µM N-(benzyloxycarbonyl) leucinylleucinylleucinal (MG-132) (Calbiochem) for the indicated times. For heat-shock HEK-293 cells were kept at 37°C as control for 24 hours or subjected to 45°C for 1 hour followed by regeneration for 23 hours at 37°C.

### Generation and transfection of expression plasmids

The cDNAs of the different CLU mRNA variants as well as Bax and Bcl-x_L_ cDNAs were cloned into expression vector pcDNA6-V5/6×His (Life Sciences). For recombinant cDNA expression 4 × 10^6^HEK-293 cells were grown in 6-well plates and transfected for 6 hours with 2 µg of plasmid DNA using OptiMem^®^ (Life Technologies) and Turbofect™ *in vitro* transfection reagent (Thermo Scientific) according to the manufacturer’s protocol. Prior to Western blot analyses transfected cells were incubated for 24 hours in serum-free medium.

### Preparation of cell lysates and Western blot analysis

Cells were lysed in ice-cold lysis buffer (50 mM Tris/HCl [pH 8], 150 mM NaCl, 1% (v/v) Triton^®^ X-100) containing protease inhibitor (Complete mini, Roche). Deglycosylation of proteins was carried out by incubation of 40 µg total protein with 1,000 units PNGase F (NEB) according to the manufacturer’s protocol. For Western blot analyses 40-150 µg of total protein or 30-40 µl of culture medium were subjected to reducing SDS-PAGE and blotted onto nitrocellulose membranes. The polyclonal antibody sc‑6419 (1:1,000 dilution, Santa Cruz) was used for detection of human CLU and monoclonal anti‑V5 antibody (Life Technologies) for detection of recombinant CLU‑V5. Human α-Tubulin was analysed via a monoclonal antibody (Sigma). Reactive bands were visualized by chemiluminescence.

### Reverse transcription, real-time PCR and 5’ RACE analyses

Total cellular RNA was isolated using the innuPrep RNA Mini Kit (Analytic Jena). Quantitative real-time PCRs were performed using 20 ng/µl of oligo dT-reverse transcribed RNA and the SYBR green / ROX based RealQ PCR Master Mix (Biomol). Quantification of mRNAs was performed in triplicates using the 7500 Fast System and SDS Software (Applied Biosystems). Plasmids carrying the respective CLU cDNAs at concentrations ranging from 10^3^-10^-5^ pg/µl served as standards for the calculation of mRNA copy numbers per ng of total RNA. For primer sequences refer to Table S1. 5’ RACE-PCR was performed according to the protocol “5’ RACE System for Rapid Amplification of cDNA Ends, Version 2.0” (Life Technologies).

### Immunocytochemistry

HEK‑293 cells were grown on coverslips (Ø 1 cm) and transfected with recombinant CLU cDNAs. If indicated, the cells were treated with 10 µM MG‑132 as described above. Paraformaldehyde-fixed cells were incubated with Alexa Fluor^®^ 488 conjugated lectins ConA or WGA (Life Technologies). After blocking cells were incubated consecutively with anti‑V5 antibody (Life Sciences) and Cy3-conjugated secondary antibody (Dianova) followed by chromatin staining with DAPI. Cells were imaged by confocal laser scanning microscopy (LSM) at a Z‑stack step size of 0.13 µm with a 63× oil immersion objective (1.4 optical aperture) using the LSM SP5 microscope (Leica) and Imaris software (Bitplane).

### Determination of caspase‑3/7 activity

1.5 × 10^4^ HEK‑293 cells were cultivated in 96-wells and transfected with 0.2 µg of plasmid DNA. After 18 hours cells were treated under serum-free conditions for 10 hours with 10 µM MG‑132 or an equivalent volume of DMSO. Caspase activity was determined using the Caspase-Glo^®^-3/7 Assay (Promega) according to the manufacturer’s protocol and a FLUOstar Omega luminometer with Omega-Data Analysis Mars software (BMG Labtech).

### NF-κB-Luciferase reporter assay

4 × 10^5^ HEK‑293 cells were cultivated in 24-wells and cotransfected with 0.3 µg of pNF-κB-Luc (Clontech) together with 0.7 µg of recombinant CLU cDNA. After 18 hours cells were treated under serum-free conditions for 24 hours with 10 ng/ml TNF-α (Sigma, diluted in 1 mg/ml BSA-solution) or an equivalent volume of 1 mg/ml BSA-solution. Cells were lysed in 100 µl Luciferase Assay Buffer (Promega). Luciferase activity was determined using the Luciferase Assay System (Promega) according to the manufacturer’s protocol and the FLUOstar Omega luminometer. Measured values were expressed relative to protein concentrations of respective wells as determined by Bradford assay. 

## Results

### Modulation of CLU mRNAs and proteins in response to proteasome inhibition

**Figure 1:**
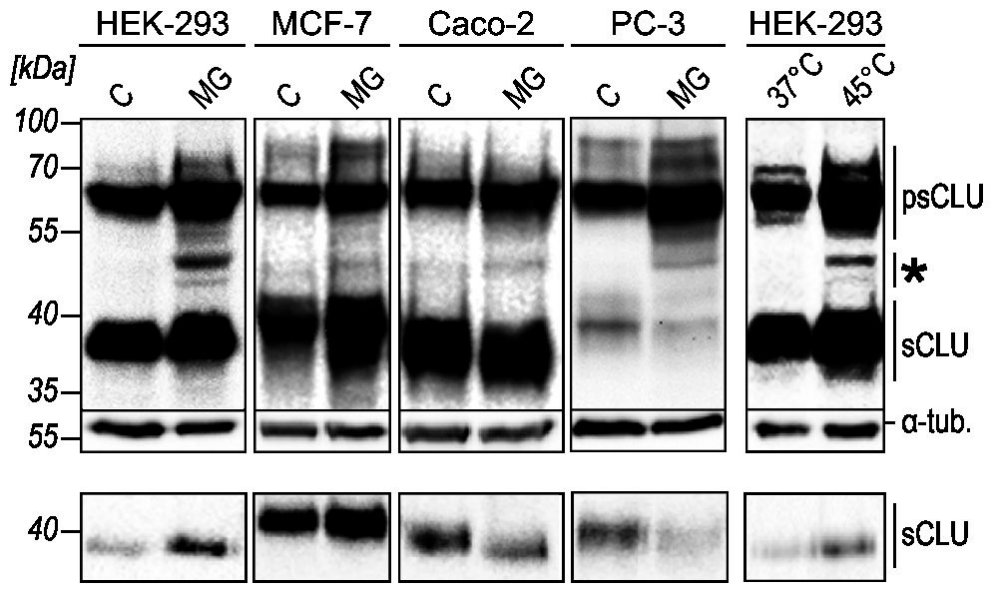
Proteasomal inhibition and heat stress modulate sCLU and intracellular CLU protein expression in cancer and non-cancer cells. HEK‑293, PC‑3, MCF‑7 and Caco-2 cells were treated with DMSO as control (C), 10 µM MG-132 (MG) or subjected to heat shock (45°C). Whole cell lysates (upper panel) and cell culture media (lower panel) of cells were analyzed for CLU expression by Western blot. 45-50 kDa CLU protein bands were detected primarily in stressed cells (*). Data shown are representative of three independent experiments.

Clusterin induction upon cell stress such as heat-shock or proteasome inhibition is a well-established phenomenon [41]. Here we focus on CLU expression in normal and in cancer cells after treatment with MG‑132, a peptide-aldehyde blocking proteasome function. First we monitored by Western blot analysis CLU expression patterns in whole cell lysates and culture supernatants of four different cell lines. Secretory CLU is synthesized as a pre-pro-protein comprising 449 amino acids (aa) with an N-terminal 22 aa signal sequence. In the ER the protein is present as a high mannose single chain precursor (psCLU) that is terminally glycosylated in the Golgi, where it is further cleaved by a furin-like protease in an N-terminal α-chain and a C-terminal β-chain. Mature sCLU is secreted as a heterodimeric protein with an apparent molecular weight (MW) of 75-80 kDa in non-reducing SDS-PAGE analyses. Under reducing conditions the α- and β-chains appear as 34-36 kDa and 37-39 kDa protein bands, respectively [55,56]. In lysates of untreated cells the antibody sc‑6419 exclusively detects psCLU (60 kDa) and the β-chain of mature sCLU (37-39 kDa). The latter is also observed in the culture medium as expected for sCLU (Figure 1). In all cell lines treatment with MG‑132 results in increased CLU protein levels in the cell lysates, however processing and secretion are modulated to various extents. Interestingly in all cell lines tested MG‑132 gives rise to additional protein bands present in minor amounts in the cell lysates: a 50 kDa band in all cell lines examined and a 45 kDa band in HEK‑293 cells. The 50 and 45 kDa bands are also observed in heat stressed HEK‑293 cells indicating that their appearance is not restricted to MG‑132 treatment but appears to be a general stress-induced phenomenon (Figure 1). The absence of these protein bands from the culture media suggests that they represent stress-induced intracellular CLU isoforms.

**Figure 2:**
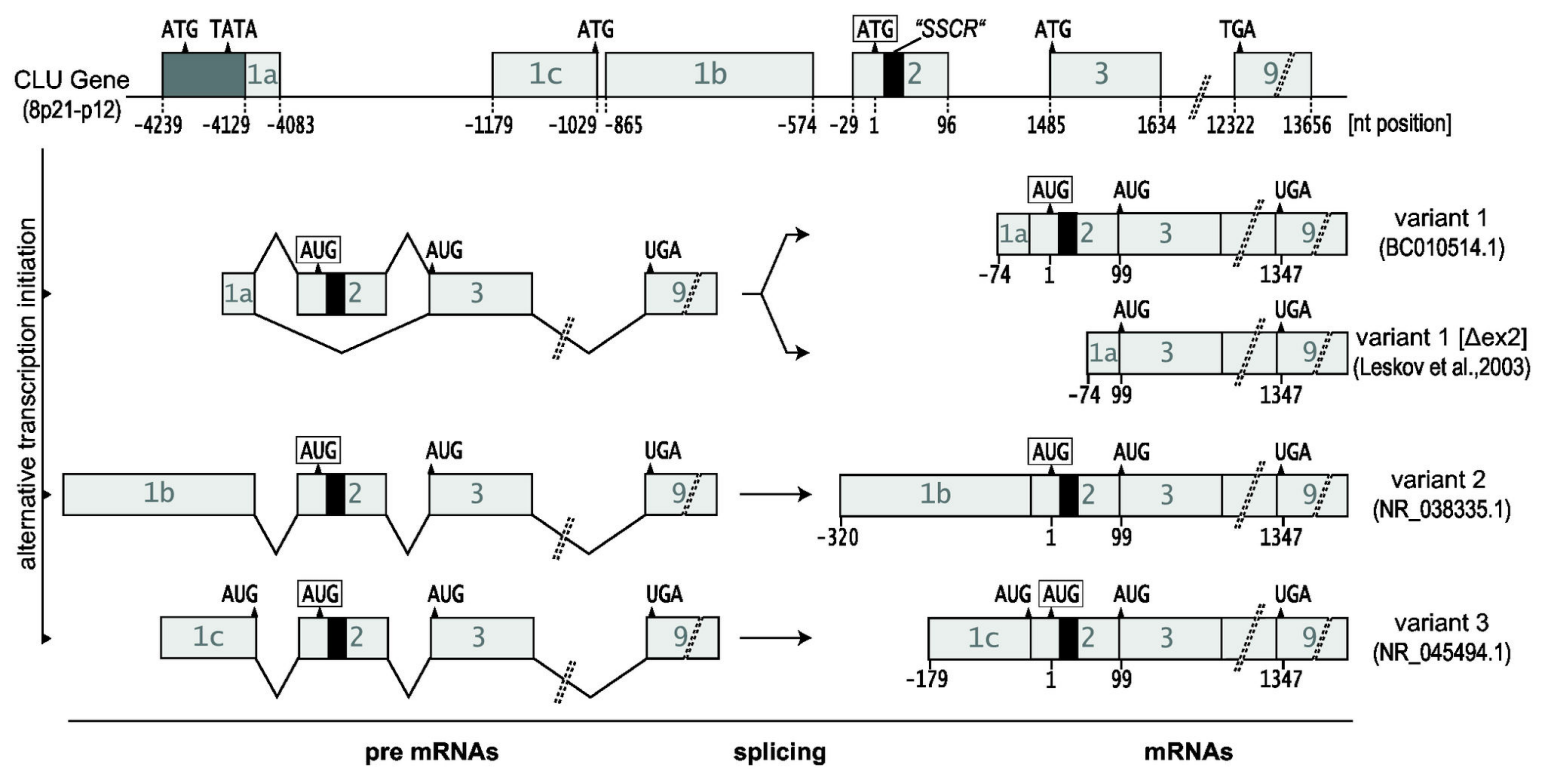
Overview of the human CLU gene and mRNA variants. The human CLU gene encodes at least 3 different pre-mRNAs which contain unique exons 1 but share exons 2-9. Alternative splicing of variant 1 pre-mRNA generates an mRNA (variant 1 [Δex2]) that lacks exon 2 and the SSCR (black box). The position of the sCLU start codon (framed) is defined as nt = 1. Notice the additional in-frame AUG codons on exon 3 of all mRNAs and on exon 1c of variant 3.

To investigate the biogenesis of these CLU forms we first analyzed the properties and expression of CLU mRNAs. The CLU gene (8p21-p12) encodes at least three different mRNA variants as currently listed in the NCBI database: variant 1 (NM_001831.3), variant 2 (NR_038335.1) and variant 3 (NR_045494.1). Since we and others revealed by 5’ RACE that the 5’-end of variant 1 differs from NM_001831.3 [14], which contains a 5’-extended exon 1 sequence, but is highly similar to the mRNA database entry BC010514.1 (Figure S1A), we hereafter refer to this sequence as mRNA variant 1. All variants are transcribed as pre-mRNAs each comprising 9 exons and 8 introns. Exon 1 sequences are unique to each of the mRNA variants arguing for distinct transcription start sites (exons 1a, 1b, and 1c). Variant 1 is known to encode sCLU that is translated from an AUG start codon located upstream of the SSCR on exon 2 (hereafter referred to as the sCLU start codon). A putative downstream in-frame start codon resides on exon 3. This codon represents the first AUG present on the previously reported alternatively spliced mRNA of variant 1 that lacks exon 2 (variant 1 [Δex2]) [38]. Translation initiation at this AUG will result in a CLU protein lacking the signal sequence, hence yielding an intracellular isoform corresponding to aa 34–449 (CLU_34‑449_) of the full-length pre-pro-protein. Variant 3 mRNA also carries a potential upstream in-frame start codon on exon 1 (Figure 2).

**Figure 3:**
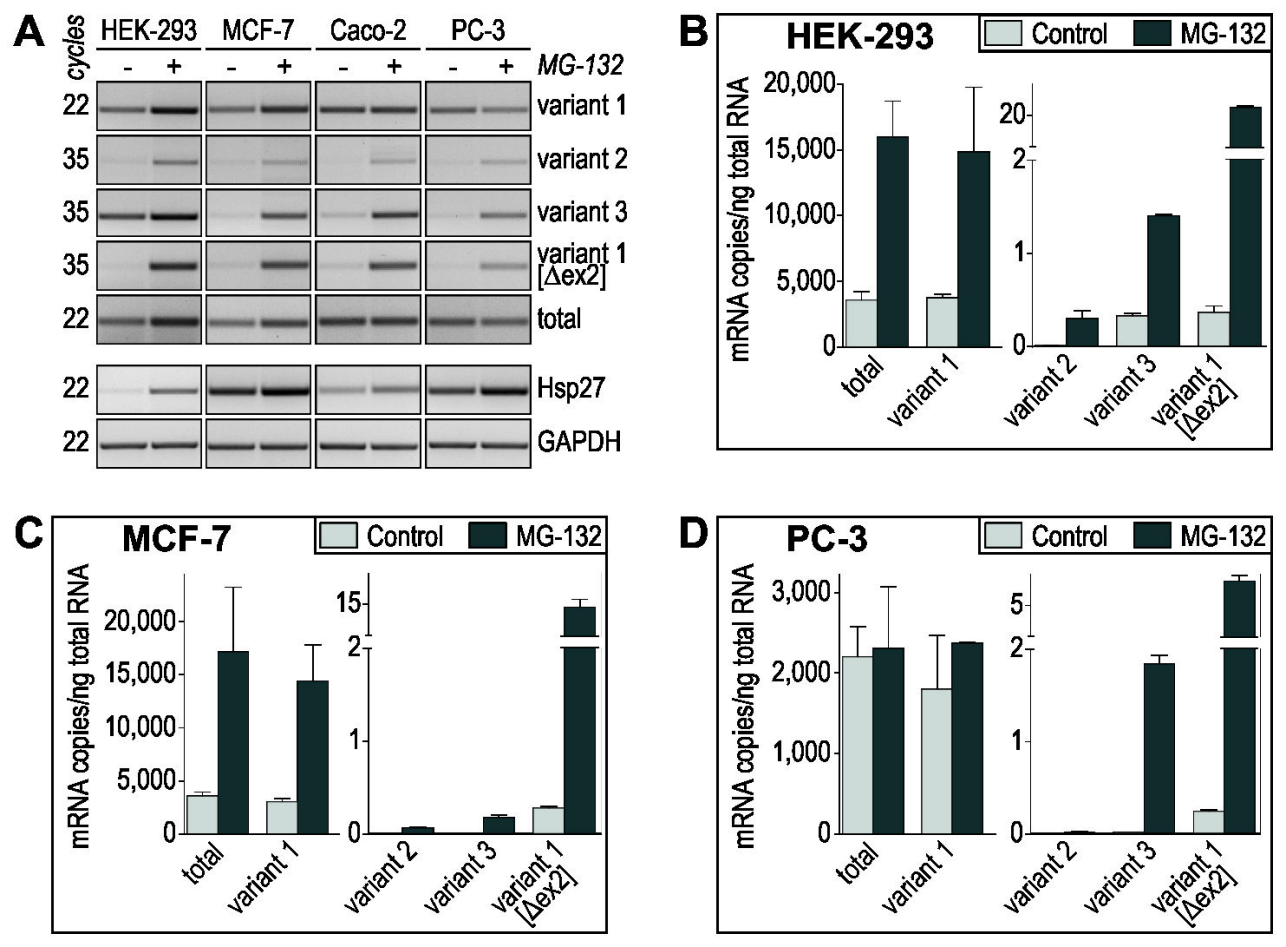
Expression of CLU mRNA variants in cancer and non-cancer cells upon proteotoxic stress. (A) Semi-quantitative RT‑PCR analyses of the expression of different CLU mRNA variants in control and MG‑132-treated HEK‑293, MCF‑7, Caco-2 and PC‑3 cells. The different numbers of amplification cycles performed reflect the varying amounts of individual CLU mRNA variants expressed. Analysis of Hsp27 mRNA indicates the induction of a heat-shock response upon MG‑132 treatment. GAPDH RT‑PCR served as control to ensure the use of equal amounts of reverse transcribed mRNA. Data shown are representative of at least 3 independent experiments. (B, C, D) Quantification of CLU mRNA expression in control and MG‑132-treated HEK‑293 (B), MCF‑7 (C) and PC‑3 cells (D) by qPCR. The amounts of individual transcripts are expressed as copy number per ng of total RNA (mean ± SD, *n* = 3). Variant 1 is the pre-dominant CLU mRNA in all cell lines conforming to the amounts of total CLU mRNA. The other variants represent low abundant CLU mRNAs.

Using variant-specific primer sets for RT‑PCR we could demonstrate the expression of all CLU mRNAs in all cell lines tested (Figure 3A). However, the copy numbers of the individual CLU mRNAs differ considerably as determined by quantitative real-time PCR (qPCR). In untreated HEK‑293 cells, variant 1 fundamentally accounts for the total amount of CLU mRNA with about 3,500 copies/ng total RNA. However, the copy numbers of variant 2 and variant 3 only add up to 0.009% of the total CLU mRNA amount. Similar ratios of CLU mRNA variants were observed in untreated MCF‑7 and PC‑3 (Figure 3B, C, *D*, light gray bars). Using specific primer sets for variant 1 [Δex2] we show that all four cell lines express this mRNA (Figure 3; Figure S1D, E), although in very low amounts (0.008%-0.01% of total CLU mRNA). Further, we detected minor expression of the 5’-extended variant 1 (NM_001831.3) in HEK‑293, MCF‑7 and Caco‑2 cells (Figure S1B, C). Incubation of the cells in presence of MG‑132 leads to an increase in CLU mRNAs variant 1 [Δex2], variant 2 and variant 3 as well as in Hsp27 mRNA (Figure 3A), the latter confirming the induction of a heat shock response. The inductions of variant 1 [Δex2], variant 2 and variant 3 range from 5- to 50-fold upon proteasome inhibition in all cells examined, yet they account for no more than 0.34% of total CLU mRNA. The copy number of the major variant 1 mRNA is increased only in HEK‑293 and MCF‑7 cells. As expected, the amount of total CLU mRNA reflects the expression level of variant 1 mRNA (Figure 3B, C, *D*, dark gray bars).

### Expression of variant specific cDNAs reveals the biogenesis of distinct CLU isoforms

**Figure 4:**
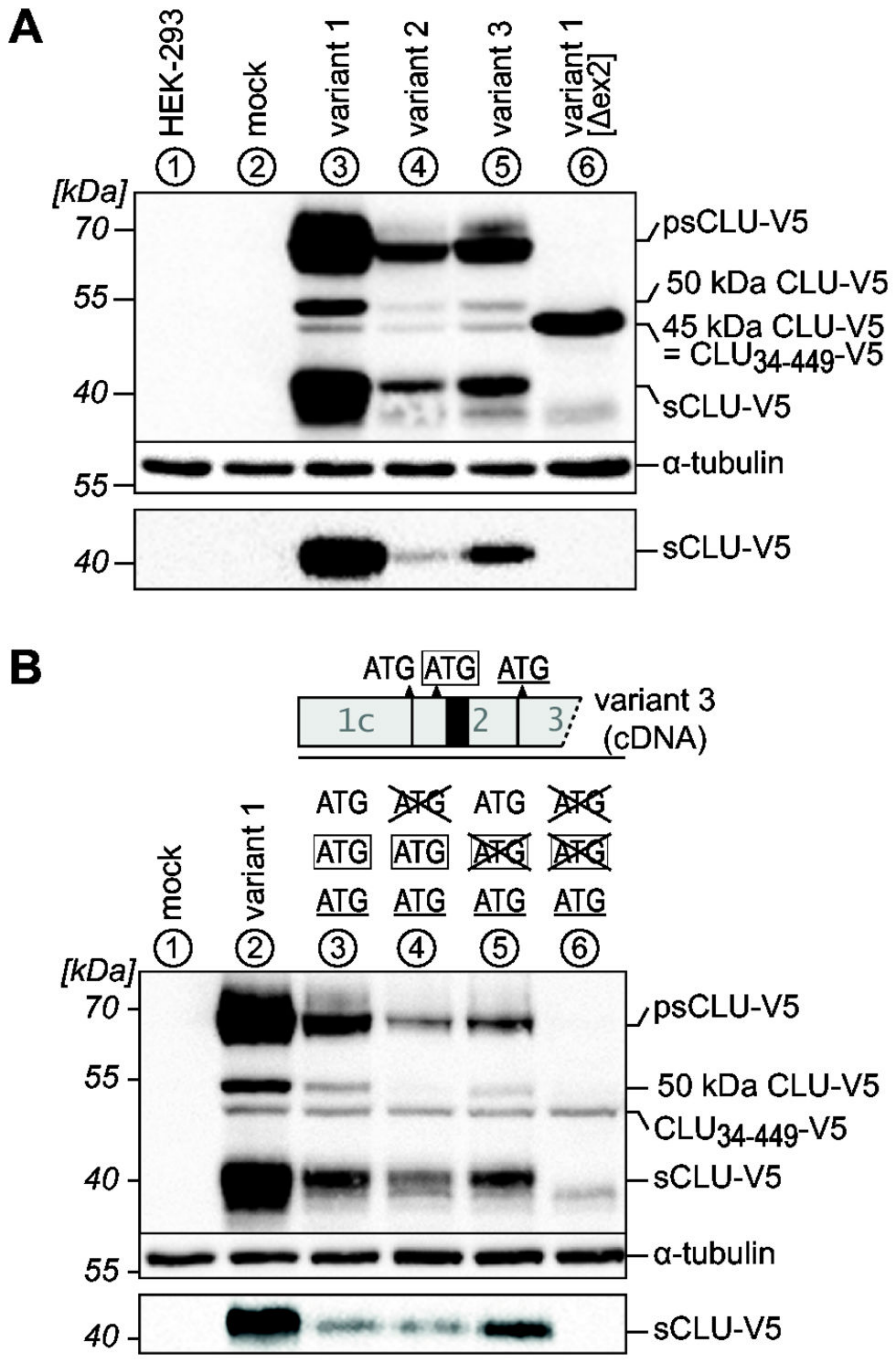
Expression of CLU-V5 proteins from recombinant cDNAs. Lysates (upper panels) and culture media (lower panels) of HEK-293 cells transiently expressing the indicated CLU cDNA variants were analyzed by Western blotting. Lanes are labeled with circled numbers. Untransfected cells (HEK‑293) or cells transfected with empty pcDNA6 (mock) served as controls. Data shown are representative of at least three independent experiments. (A) Transfection of cDNA variants 1, 2 and 3 leads to expression and secretion of sCLU (lanes 3-5). Variant 1 [Δex2] produces a non-secreted 45 kDa CLU‑V5 protein corresponding to CLU_34‑449_ (lane 6). This form is also present in low amounts in the lysates of cells transfected with the cDNA variants 1, 2 and 3. Furthermore, cells transfected with these variants express an additional non-secreted 50 kDa CLU‑V5 protein. (B) A schematic outline of the 5’-end of cDNA variant 3 is shown. Neither point-mutations (crossed out codons) of the sCLU start codon (framed, lane 5) nor the in-frame ATG on exon 1c (lane 4) completely block sCLU expression. Concurrent mutation of both codons eliminates sCLU synthesis (lane 6).

We then aimed to characterize the biogenesis of the distinct CLU forms by overexpressing cDNA constructs of all mRNA variants. To differentiate between endogenously and ectopically expressed CLU forms the recombinant proteins were tagged with a 5 kDa C-terminal V5-epitope (hereafter abbreviated “‑V5”). We chose HEK‑293 cells for these experiments, as they endogenously express all mRNA variants and should therefore be capable to correctly synthesize all recombinant CLU forms. Transfection of CLU cDNAs variant 1, 2 and 3 which contain exon 2, leads to the synthesis and secretion of sCLU (Figure 4A, lanes 3-5). Although these variants are expressed from recombinant DNA under the control of the CMV promotor sCLU expression from variant 1 cDNA vastly exceeds the amounts synthesized from variant 2 and variant 3 cDNAs. As shown by *in vitro* mutagenesis this is attributed to out of frame upstream open reading frames (uORFs) on variant 2 and 3 mRNAs interfering with translation initiation at the sCLU start codon (Figure S2A). Variant 3 mRNA contains an additional upstream in-frame AUG codon on exon 1. It has been speculated that expression from this codon would lead to intracellular CLU by suppressing the function of the signal sequence [36]. However, in *vitro* mutagenesis of the exon 1 ATG and/or the sCLU start codon on variant 3 cDNA revealed that usage of both start codons results in the synthesis of sCLU (Figure 4B), thus demonstrating that expression from the upstream ATG may occur and does not suppress signal sequence function. Similar results were obtained for the upstream in frame ATG on exon 1 of the 5’ extended variant 1 (NM_001831.3) (Figure S2B).

**Figure 5:**
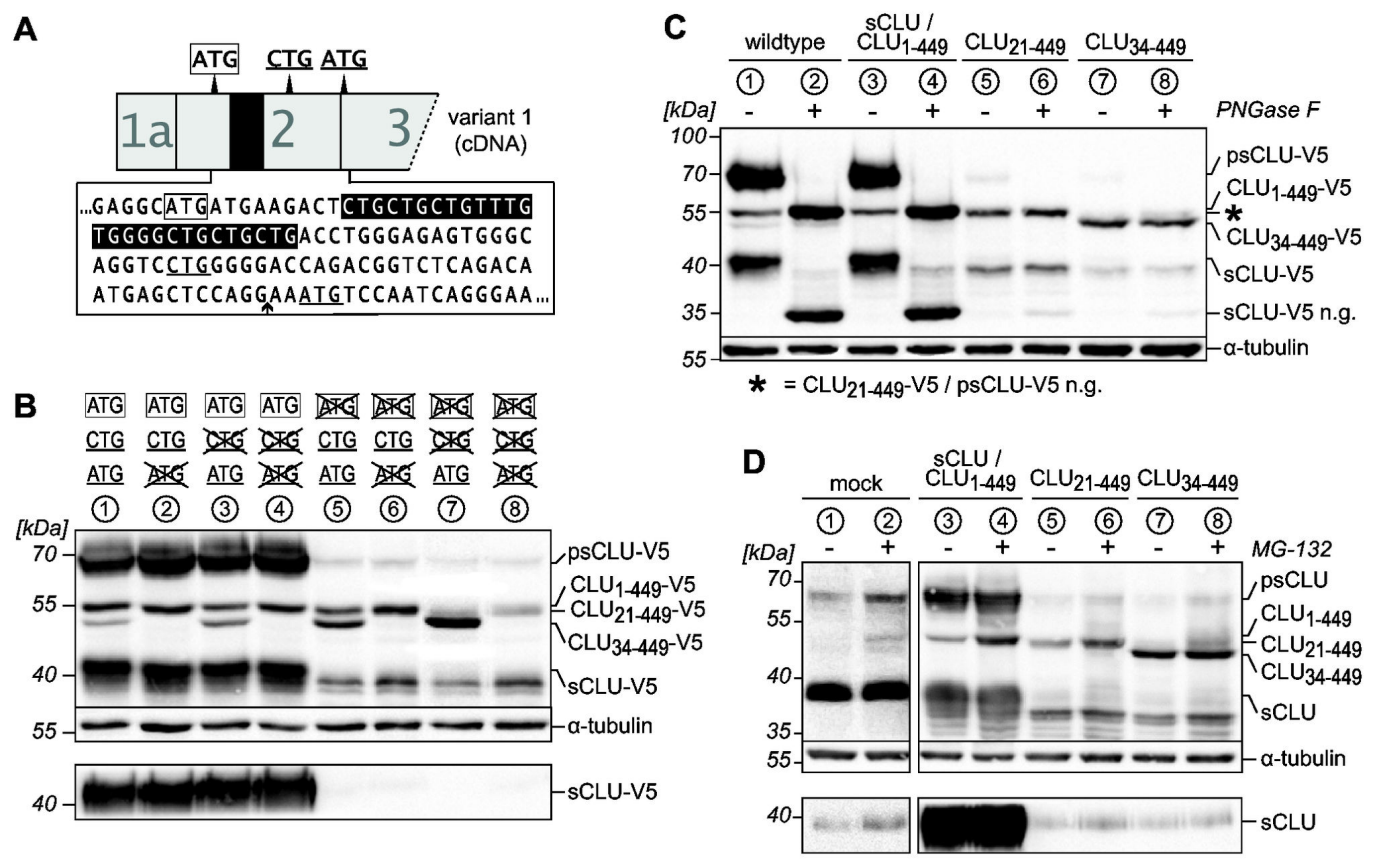
Characterization of CLU-isoform biogenesis. (A) Schematic outline of the 5’-sequence of variant 1 showing the sCLU start codon (framed) and the downstream start codon on exon 3 (underlined). A non-canonical CTG start codon is present on exon 2 (underlined). The SSCR (black shaded nucleotides) and the exon 2/exon 3 border (arrow) are indicated. (B) Western blots of recombinant CLU-V5 proteins in lysates (upper panel) and culture media (lower panel) of HEK-293 cells transiently expressing unmodified or point-mutated (crossed out codons) CLU cDNA variant 1. CLU_34‑449_ is translated from the ATG codon on exon 3 (lanes 2, 7). The 50 kDa CLU‑V5 band consists of the sCLU pre-pro-protein (CLU_1‑449_) translated from the sCLU start codon and CLU_21‑449_ translated from the CTG codon (lanes 4, 6). (C) Western blot of recombinant CLU-V5 proteins in lysates of HEK-293 cells transiently expressing sCLU/CLU_1‑449_, CLU_21‑449_ or CLU_34‑449_ from point-mutated variant 1 cDNAs or unmodified variant 1 cDNA (wildtype). Lysates were treated with PNGase F as indicated. The molecular weights of psCLU and sCLU decrease upon deglycosylation (psCLU/sCLU n.g., lanes 3, 4). PNGase F treatment does not alter the molecular weights of CLU_1‑449_ (lanes 3, 4), CLU_21‑449_ (lanes 5, 6) and CLU_34‑449_ (lanes 7, 8). (D) Western blots of untagged CLU proteins in lysates (upper panel) and culture media (lower panel) of control and MG-132-treated HEK-293 cells transiently expressing sCLU/CLU_1‑449_, CLU_21‑449_ or CLU_34‑449_ from point-mutated variant 1 cDNAs or transfected with pcDNA (mock). In contrast to CLU_1‑449_ and CLU_21‑449_ which accumulate upon proteasome inhibition (lanes 3-6), the amount of CLU_34‑449_ is not affected (lanes 7, 8). (B, C, D) Data shown are representative of three independent experiments. Lanes are labeled with circled numbers. Recombinant CLU protein bands with a molecular weight of ~38 kDa presumably originate from even further downstream translation initiation sites on CLU cDNAs.

Cells transfected with variant 1 [Δex2] cDNA do not secrete recombinant sCLU but express a 45 kDa CLU‑V5 protein in the lysates (Figure 4A, lane 6). Notably, an intracellular 45 kDa CLU‑V5 protein is also expressed, although to a lesser extent, from variant 1, 2 and 3 cDNAs (Figure 4A, lanes 3-5). As shown by *in vitro* mutagenesis, translation of this protein initiates at the downstream in-frame ATG on exon 3 (Figure 5B, lanes 2, 4, 6, 8). Therefore the 45 kDa CLU form observed in stressed HEK‑293 cells could arise from variant 1 [Δex2] CLU mRNA and/or internal translation initiation on exon 3 of variant 1, 2 and 3 CLU mRNAs and represents the isoform CLU_34‑449_.

With regard to the endogenous 50 kDa CLU protein band observed within stressed cells it is interesting that a 50 kDa CLU‑V5 form cannot be detected upon expression of variant 1 [Δex2] cDNA but is exclusively synthesized from cDNA variants 1, 2 and 3 (Figure 4A, lanes 3-5). To elucidate the nature of this CLU protein, we asked: 1) whether it could originate from still unknown in-frame start codons on exon 2 downstream the sCLU start codon and/or 2) whether it represents unglycosylated sCLU pre-pro-protein that has not been translocated into the ER. In support of possibility 1, inactivation of the sCLU start codon on variant 1 abrogates sCLU synthesis, but does not impair expression of the 50 kDa CLU‑V5 protein (Figure 5B, lane 5). Therefore we proposed a CTG codon surrounded by an adequate Kozak sequence on exon 2 (Figure 5A, underlined) as an unconventional translation initiation site. Indeed, point-mutation of this CTG codon on a cDNA carrying an inactivated sCLU start codon strongly inhibits the expression of the 50 kDa CLU‑V5 form, demonstrating translational initiation at this site (Figure 5B, lane 7). However, after transfection of a cDNA containing exclusively the sCLU start codon as active translational start site, apart from sCLU also significant amounts of a 50 kDa CLU‑V5 protein are expressed (Figure 5B, lane 4). This indicates that the 50 kDa CLU band actually represents two distinct CLU proteins with a similar apparent molecular weight in SDS-PAGE analyses. One is translated from the proposed CTG codon and corresponds to aa 21–449 (CLU_21‑449_) therefore lacking the SSCR. Since the other 50 kDa CLU protein depends on translational initiation at the sCLU start codon it could well represent sCLU pre-pro-protein which is not segregated into the ER as proposed above (CLU_1‑449_). To scrutinize this notion we treated lysates obtained from HEK‑293 cells overexpressing sCLU–V5/CLU_1‑449_-V5, CLU_21‑449_-V5 or CLU_34‑449_-V5 with PNGase F. The molecular weights of CLU_21‑449_-V5 and CLU_34‑449_-V5 proteins remain unaffected by deglycosylation demonstrating that these CLU forms do not contain any polysaccharide moieties (Figure 5C, lanes 5-8). As expected, deglycosylation leads to a drop in molecular weight of psCLU-V5 and sCLU-V5 to 50 kDa and 35 kDa respectively. However, no additional bands, which would correspond to deglycosylated CLU_1‑449_-V5 are observed after PNGase F-treatment indicating an unglycosylated state of CLU_1‑449_ (Figure 5C, lanes 3, 4).

### Post-translational mechanisms contribute to the accumulation of both 50 kDa CLU isoforms, but not the 45 kDa CLU isoform in MG-132-treated cells

After having revealed the origin of the intracellular CLU isoforms generated within unstressed and stressed cells, we investigated whether in addition to transcriptional upregulation also post-translational mechanisms (i.e. reduced proteasomal degradation) contribute to the MG‑132‑induced accumulation of endogenous intracellular CLU forms. We therefore overexpressed sCLU/CLU_1‑449_, CLU_21‑449_ and CLU_34‑449_ as untagged proteins under control of the constitutive CMV-promotor followed by treatment of the cells with MG‑132. By this experimental design we could exclude the involvement of transcriptional regulation in the accumulation of CLU proteins and exactly align recombinantly with endogenously expressed CLU bands. When exclusively sCLU is expressed, MG‑132 treatment does not affect the amounts of psCLU and mature sCLU but selectively leads to an accumulation of CLU_1‑449_, which comigrates with the endogenous 50 kDa protein band detected in stressed mock-transfected cells (Figure 5D, lanes 3, 4). Likewise, we observed an MG‑132-induced accumulation of recombinant CLU_21‑449_, but not of recombinant CLU_34‑449_, which comigrates with the endogenous 45 kDa CLU form observed in MG‑132-treated mock-transfected cells (Figure 5D, lanes 5-8). These results strengthen the idea that the endogenous 50 kDa CLU protein expressed in various cells after proteasome inhibition corresponds to CLU_1‑449_ and/or CLU_21‑449_ and, that an impaired proteasomal degradation contributes to the accumulation of these CLU form(s) within stressed cells. In contrast, upregulation of the endogenous 45 kDa CLU form in MG‑132-treated HEK‑293 cells seems to occur exclusively on the transcriptional level probably by induction of CLU mRNAs variant 1 and variant 1 [Δex2].

### CLU_21–449_ and CLU_34–449_ are located in the cytosol of unstressed and stressed cells

To track the subcellular localization of the different CLU forms under physiologic conditions and upon proteotoxic stress we expressed these as V5-tagged proteins. After transfection of corresponding CLU cDNAs in HEK‑293 cells we performed immunocytochemistry followed by laser scanning microscopy (LSM).

**Figure 6:**
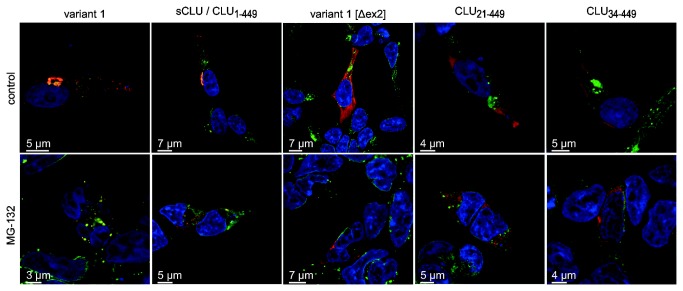
Subcellular localization of individual CLU isoforms. HEK‑293 cells were transfected with unmodified variant 1, variant 1 [Δex2] or point-mutated versions of variant 1 cDNA encoding only sCLU/CLU_1‑449_, CLU_21‑449_ or CLU_34‑449_ and subjected to LSM. CLU-V5 was detected by the anti‑V5 primary antibody and the Cy3-conjugated secondary antibody (red). Alexa Fluor^®^ 488-conjugated ConA (green) and DAPI (blue) served as counterstains for the nuclear membrane-ER continuum and the nucleus, respectively. Images shown represent the middle plane of the analyzed cells. When unmodified variant 1 cDNA or sCLU/CLU_1‑449_ are expressed the staining of CLU and ConA shows an overlay (yellow) caused by the presence of psCLU in the ER (variant 1, sCLU/CLU_1‑449_, control). Expression of variant 1 [Δex2] leads to a mutual exclusive CLU and ConA staining (variant 1 [Δex2], control). A similar staining is observed for CLU_21‑449_ and CLU_34‑449_ (CLU_21‑449_, CLU_34‑449_, control). The presence of 10 µM MG‑132 does not lead to alterations in the subcellular localization of the individual CLU isoforms when compared to untreated controls. The disruption of intracellular membranes, condensed chromatin and nuclear fragmentation is indicative for apoptotic processes induced by MG‑132 treatment.

In unstressed cells, expression of variant 1 cDNA results in a vesicular and perinuclear CLU staining. As expected for sCLU, which is the major form expressed from variant 1 cDNA (Figure 4A), CLU staining colocalizes with both, Concanavalin A (ConA) and wheat germ agglutinin (WGA) staining, indicating its localization in the ER and the Golgi compartments (Figure 6, Figure S3; variant 1 control). CLU_1‑449_, CLU_21‑449_ and CLU_34‑449_ which are also synthesized in small amounts upon expression of variant 1 are not detectable in these samples due to the overwhelming sCLU and psCLU staining. We obtained identical results when the cells were transfected with point-mutated variant 1 cDNA carrying only an active sCLU start codon (Figure 6, Figure S3; sCLU / CLU_1‑449_ control). In contrast, after transfection with variant 1 [Δex2] cDNA or modified variant 1 cDNA encoding only CLU_34‑449_ the resulting CLU fluorescence shows no overlay with ConA, WGA or DAPI staining but an almost even distribution throughout the rest of the cell that is characteristic of a soluble cytoplasmic protein (Figure 6, Figure S3; variant 1 [Δex2] control, CLU_34‑449_ control). The same applies to the subcellular localization of CLU_21‑449_ in unstressed cells (Figure 6, Figure S3; CLU_21‑449_ control).

Upon incubation with MG‑132, the number of cells rounding up and detaching from the culture surface increased. In LSM analyses these cells appear shrunken with nuclear fragmentation and disruption of intracellular compartments like ER and Golgi, which is indicative of advanced stages of apoptosis. Concomitantly, sCLU perinuclear staining becomes spotty throughout the cytoplasm (Figure 6, Figure S3; variant 1 MG‑132, sCLU / CLU_1‑449_ MG‑132). In contrast, CLU_21‑449_ and CLU_34‑449_ keep their cytosolic localization after MG‑132 treatment. However, the slightly spottier distribution of their fluorescence indicates the formation of CLU_21‑449_- and CLU_34‑449_-containing protein aggregates upon proteotoxic stress (Figure 6, Figure S3; variant 1 [Δex2] MG‑132, CLU_21‑449_ MG‑132, CLU_34‑449_ MG‑132). In none of these experiments, we were able to detect an unambiguous nuclear localization of CLU. In rare cases we observed individual cells displaying an apparent nuclear spotty CLU staining (Figure S3). Detailed analysis of these cells by animated Z‑stacks of the LSM data revealed that these CLU spots are caused by cytoplasmic/cytosolic invaginations into the nuclear compartment rather than representing a localization of CLU in the nucleoplasm (Video S1).

### Distinct CLU isoforms do not affect caspase‑3/7-mediated apoptosis or NF-κB-activity

**Figure 7:**
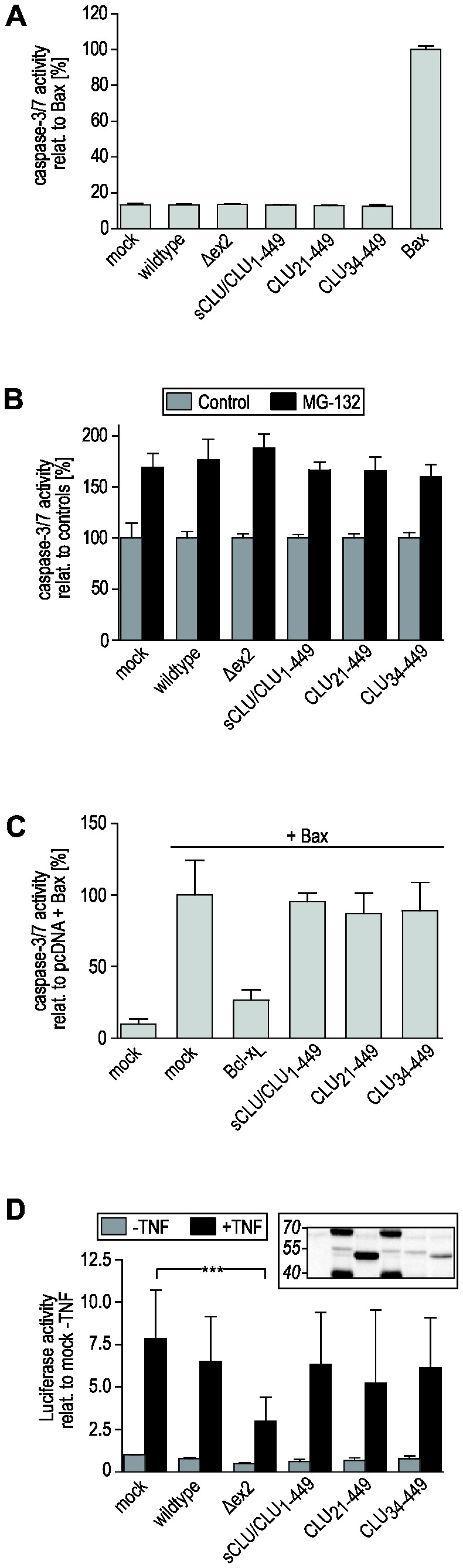
Impact of individual CLU isoforms on apoptosis and on NF-κB-activity. HEK‑293 cells were transfected with pcDNA6 (mock), unmodified variant 1 (wildtype), variant 1 [Δex2] (Δex2) or point-mutated versions of variant 1 cDNA encoding only sCLU/CLU_1‑449_, CLU_21‑449_ or CLU_34‑449_. (A) 24 hours after transfection the activity of caspases 3 and 7 was determined. Data are expressed relative to the values obtained from Bax cDNA transfected cells (mean ± SD, n = 3), which served as a positive control. In contrast to Bax, the expression of all CLU protein forms does not activate caspase‑3/7. (B) 24 hours after transfection 10 µM MG‑132 or DMSO (control) was added for 10 hours followed by measurement of caspase 3/7 activity. Data are expressed relative to the values obtained from corresponding control cells (mean ± SD, n = 3). MG‑132 treatment causes an increase in caspase 3/7 activity in all cells analyzed. Neither CLU protein form significantly affects the extent of MG‑132-induced caspase‑3/7 activation. (C) HEK-293 were cotransfected with Bax cDNA together with pcDNA6 (mock +Bax), Bcl-x_L_ or point-mutated versions of variant 1 cDNA. 24 hours after transfection the activity of caspases 3 and 7 was determined. Cells transfected with pcDNA6 alone (mock) served as negative control. Data are expressed relative to the values obtained from mock +Bax transfected cells (mean ± SD, n = 3). While cotransfection with Bcl-x_L_ cDNA, suppresses caspase 3/7 activation demonstrating the validity of the experimental setup, neither of the CLU isoforms significantly modulates Bax-mediated apoptosis. (D) HEK-293 were cotransfected with pNF-κB-Luc and the indicated versions of variant 1. 18 hours after transfection cells were incubated for 24 hours with either 10 ng/ml TNF-α (+TNF) or with BSA solution (-TNF). Cells were lysed and luciferase activity was determined as a measure of NF-κB-activity. Data are expressed as increase in Luciferase activity relative to mock transfected cells incubated with BSA solution (mean ± SD, n=4). A representative Western Blot of cell lysates is embedded showing CLU expression in the samples analyzed. The order is: mock, wildtype, Δex2, sCLU/CLU_1‑449_, CLU_21‑449_ and CLU_34‑449_. Only CLU_34‑449_ expressed from variant 1 [Δex2] reduces NF-κB activity (Δex2).

The ability to express distinct CLU isoforms independently allowed us to analyze their impact on cellular processes in which the function of CLU isoforms is intensively discussed. In apoptosis, intracellular CLU isoforms have been reported to act as pro-death factors [34,38]. Therefore we first of all investigated whether overexpression of individual CLU isoforms is sufficient to promote caspase‑3/7 activation. Compared to overexpressed Bax, which served as a positive control, neither expression of sCLU/ CLU_1‑449_, CLU_21‑449_ nor CLU_34‑449_ increases caspase 3/7 activity within unstressed HEK‑293 (Figure 7A) and PC‑3 cells (Figure S4). Since CLU is further suggested to mediate MG-132-induced apoptosis [54] we next investigated the effect of individual CLU isoforms on the extent of caspase‑3/7 activity in MG-132-treated cells. As expected, MG‑132 induces caspase 3/7 activity in mock transfected HEK‑293 cells. Upon overexpression of individual CLU isoforms, however, we observed no significant differences in the extent of MG-132-induced caspase 3/7 activity (Figure 7B) arguing against a CLU-isoform specific modulation of MG-132-induced apoptotic processes. In this context the role of CLU in the regulation of Bax-function is debated since it has been reported to promote as well as to inhibit Bax-mediated intrinsic apoptosis [41,42,43]. In contrast to B-cell lymphoma 2-like 1 (Bcl-x_L_), a Bax-antagonizing anti-apoptotic protein that was used as positive control, neither sCLU/CLU_1‑449_, CLU_21‑449_ nor CLU_34‑449_ affected caspase 3/7 activation when coexpressed with Bax in HEK‑293 cells (Figure 7C). In summary, we could not detect any pro- or anti-apoptotic functions under normal and stress conditions of either the secretory or the non-secreted intracellular CLU forms.

The regulation of NF-κB-activation is another proposed function of CLU. However, both, NF-κB-stimulatory and -inhibitory properties have been described [44,45], which might be attributed to different CLU isoforms. By using an NF-κB-controlled Luciferase reporter plasmid, we determined the impact of individual CLU isoforms on the TNF-α-induced NF-κB activity. Incubation with TNF-α leads to an 8-fold increase in NF-κB activity in HEK-293 cells cotransfected with pNF-κB-Luc and pcDNA6 (Figure 7D, mock). Neither expression of unmodified variant 1, of sCLU/CLU_1‑449_ nor of CLU_21‑449_ does affect TNF-α-induced NF-κB activity. The same is observed for CLU_34‑449_ when being expressed from point-mutated variant 1 (Figure 7D, CLU_34‑449_). However, the latter isoform reduces NF-κB activity when being expressed from variant 1 [Δex2] (Figure 7D, Δex2) which might reflect the higher amount of CLU_34‑449_ expressed from this cDNA.

## Discussion

In this study we have addressed an issue that has engaged the interest of researchers for a long time; namely the regulation and function of distinct CLU mRNAs and protein isoforms during proteotoxic stress. Here we could show for the first time, that translation of all exon 2-containing CLU mRNAs (BC010514.1, NR_038335.1, NR_045494.1, NM_001831.3) leads to predominant sCLU synthesis. Contrary to previous suggestions [36,57] our data demonstrate that in addition to the sCLU start codon, the initiation of sCLU translation may also occur at in-frame AUGs on exon 1 of variant 3 and the 5’-extended version of variant 1 (NM_001831.3). However, variant 1 (BC010514.1) is the dominant CLU mRNA contributing to the vast majority of extracellular sCLU protein. Variant 2 and variant 3 mRNAs represent low-abundant CLU mRNAs with suppressed sCLU synthesis due to interfering uORFs on their exon 1 sequences. Hence, the translational contribution of these variants to total CLU protein amount is insignificant, therefore challenging their physiological relevance.

**Figure 8:**
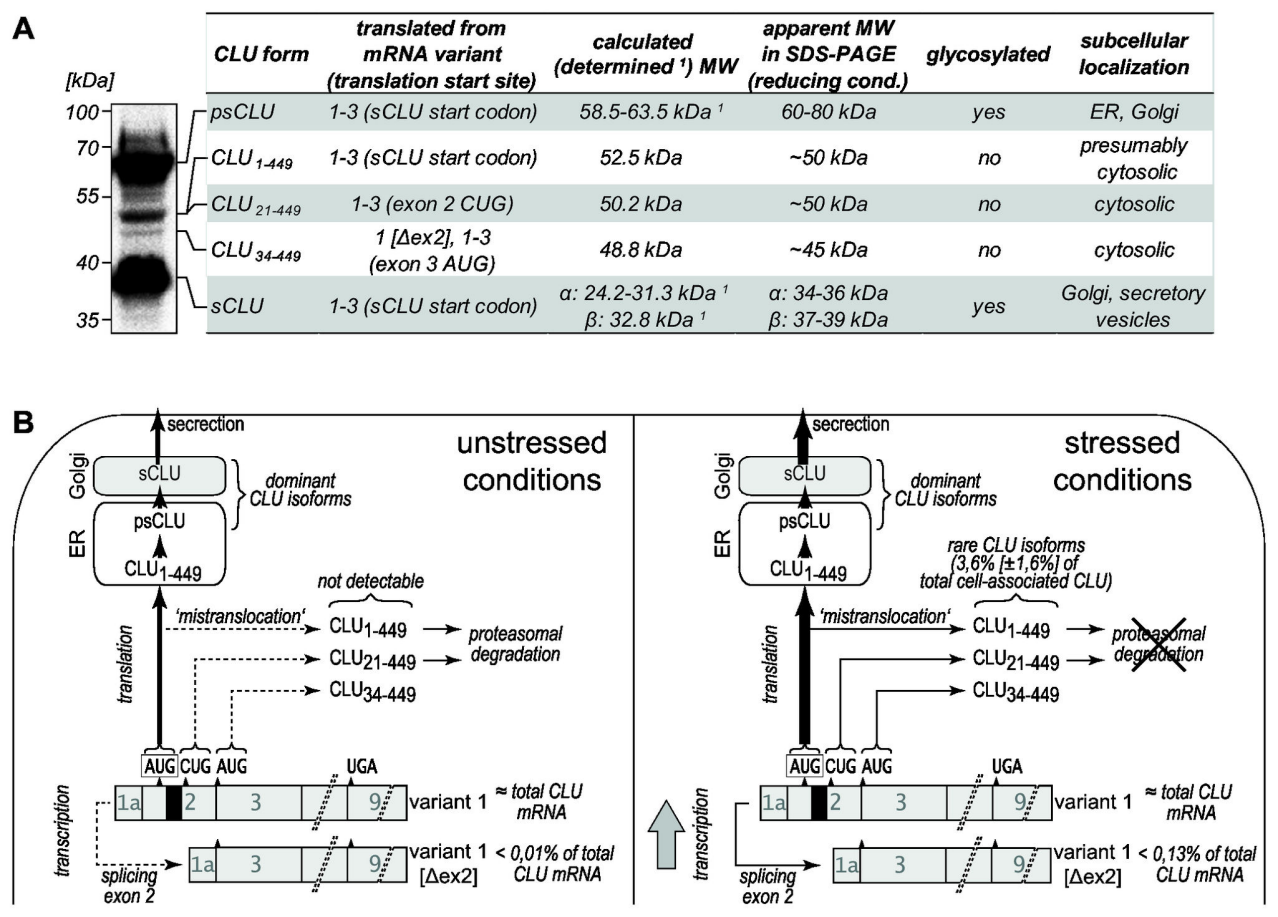
Properties of distinct CLU isoforms and their synthesis in unstressed and stressed cells. (A) A CLU-specific Western blot of cell lysate of MG‑132-treated HEK‑293 cells is shown. All detectable protein bands can be assigned to distinct CLU forms with different properties and subcellular localization (^1^ determined MWs of secretory CLU forms according to Kapron et al. [55]). (B) Model depicting the synthesis of CLU isoforms in unstressed and stressed HEK-293 cells. Under unstressed conditions, the dominant isoform sCLU is translated from variant 1, which accounts virtually for the total CLU mRNA amount. Cytosolic CLU_1‑449_ (‘mistranlocated’ sCLU pre-pro-protein) and CLU_21‑449_ (translated from exon 2 CUG) are not detectable due to presumably low expression and proteasomal degradation. CLU_34‑449_ is translated from exon 3 AUG on variant 1 and from variant 1 [Δex2]. Although CLU_34‑449_ is not proteasomally degraded, it is not detectable in unstressed cells reflecting its low expression level. Induction of cellular stress (MG-132, heat) induces transcriptional upregulation of variant 1 and its splicing to variant 1 [Δex2] leading to enhanced synthesis of all CLU isoforms. Further, the ‘mistranslocation’ of CLU_1‑449_ to the cytosol is increased. In the case of proteotoxic stress induced by MG-132, diminished proteasomal degradation of CLU_1‑449_ and CLU_21‑449_ further promotes their accumulation. Altogether these events generate amounts of the rare cytosolic CLU isoforms that are detectable in Western Blot analyses. They account for 3.6% ± 1.6% (mean ± SD, *n*=5) of total cell-associated CLU in stressed cells as determined by densitometric quantification of corresponding bands. Note that different expression levels (from low to high) are indicated by the different line width of arrows (from dashed to bold). Variants 2 and 3 are not illustrated because they are expressed in very low amounts and virtually do not contribute to the synthesis of any CLU isoform.

In addition to modulating sCLU expression, cellular stress induces the accumulation of non-glycosylated cytosolic 50 and 45 kDa CLU isoforms (Figure 8A). Here we show that the former actually consists of two distinct proteins translated from variant 1 mRNA: One protein represents unglycosylated sCLU pre-pro-protein (CLU_1‑449_) that is not translocated into the ER lumen under stress conditions. Very recently, the existence of this CLU isoform has been demonstrated in the cytosol of HeLa cells [58]. Remarkably, similar observations have been made for major prion protein (PrP) that is normally cotranslationally segregated across the ER membrane. However, ER stress favors the ‘mistranslocation’ of a PrP isoform still carrying the signal sequence to the cytosol, where it accumulates as a potentially cytotoxic protein [59]. The other 50 kDa CLU protein, CLU_21‑449,_ is generated, by unconventional translation from a well-conserved CUG codon located on exon 2 resulting in an unglycosylated isoform lacking the signal sequence. Interestingly, 10 nucleotides surrounding this CUG codon show a high degree of homology to a CUG translation initiation site found in the internal ribosome entry site of human fibroblast growth factor 2 [60] suggesting that a similar functional sequence on exon 2 of CLU mRNAs could lead to the expression of cytosolic CLU_21‑449_. The 45 kDa CLU protein represents yet another cytosolic and non-glycosylated CLU isoform lacking the signal sequence (CLU_34‑449_). It is generated from alternatively spliced variant 1 mRNA in which exon 2 has been removed. Thus far, this mRNA variant 1 [Δex2] has only been reported for gamma-irradiated MCF-7 mammary gland carcinoma cells [39]. We here demonstrate for the first time that this mRNA is present in other cell types, most intriguingly even under normal conditions (HEK-293 cells). Stress-induced alternative mRNA splicing is well-known. It involves canonical splicosome dependent and unconventional cytosolic mechanisms [61,62], thereby increasing the diversity or shifting the balance between stress-related protein isoforms. Interestingly, similar to CLU_1‑449_ and CLU_21‑449_ minor amounts of CLU_34‑449_ are also synthesized from non-spliced variant 1 mRNA. This supports previous publications which proposed internal initiation at the exon 3 AUG on CLU mRNA, contributing to CLU_34‑449_ synthesis [34,35]. We here show that in HEK-293, MCF-7 and Caco-2 cells variant 1 mRNA is induced upon MG-132 treatment. This is in accordance to previous studies demonstrating regulation of CLU mRNA by heat-shock factors (HSFs) which bind to the CLE motif within the CLU promotor [18,63]. Thus, the increased sCLU/CLU_1‑449_, CLU_21‑449_ and in part CLU_34‑449_ expression are attributed to upregulation of variant 1 within stressed cells.

Our data further reveal that in contrast to CLU_1‑449_ and CLU_21‑449_, which accumulate upon proteasome inhibition, CLU_34‑449_ is not subjected to proteosomal degradation. Thus, we suggest that under conditions of impaired proteasomal activity, expression of CLU_34‑449_ is regulated exclusively on the transcriptional level by increased synthesis of variant 1 and variant 1 [Δex2] mRNA, while reduced proteasomal degradation clearly contributes to the accumulation of the endogenous 50 kDa isoforms CLU_1‑449_ and CLU_21‑449_ [54,63]. These cytosolic proteins represent rare CLU isoforms altogether accounting for less than 5% of total cell-associated CLU in stressed cells (Figure 8B). Recently, hypoglycosylated psCLU (55-60 kDa) after retrotranslocation from the ER has been reported to represent an additional cytosolic CLU isoform [32,33]. In support of this, we observed a CLU protein band of ~55 kDa in MG-132-treated HEK-293 cells that presumably corresponds to hypoglycosylated CLU (Figure 1, Figure 5D, lane 4, Figure 8A).

In summary, alternative splicing (CLU_34‑449_), internal translation initiation (CLU_21‑449_, CLU_34‑449_), ‘mistranslocation’ of sCLU pre-pro-protein (CLU_1‑449_) as well as impaired proteasomal degradation (CLU_1‑449_, CLU_21‑449_) contribute to the biogenesis of cytosolic CLU isoforms. The properties of all individual CLU isoforms are summarized in Figure 8A. Previous works describe a 50 kDa CLU protein band as a post-translationally modified, “activated” form of CLU_34‑449_ [38,39]. Our results, however, demonstrate that the 50 kDa CLU forms (CLU_1‑449_ and CLU_21‑449_) as well as CLU_34‑449_ are all independently synthesized proteins.

The subcellular localization of intracellular CLU has been studied previously by immunofluorescence microscopy. We could confirm the presence of psCLU/sCLU in the ER/Golgi continuum [33,64] and the cytosolic distribution of CLU_34‑449_ [43,65,66]. However, no nuclear localization of CLU, even in late-stage apoptotic cells was detectable. This does not support previous reports suggesting translocation of CLU_34‑449_ into nucleus under stress conditions [38,54,67,68]. Furthermore, considering the low endogenous amounts of CLU_1‑449_, CLU_21‑449_ and CLU_34‑449_ (compared to psCLU/sCLU) present within stressed cells, it has to be concluded that none of these cytosolic isoforms can realistically account for the often reported major stressed-induced changes in subcellular distribution of CLU in cells.

The function of distinct CLU isoforms in intrinsic apoptosis and NF-κB-mediated signaling is unclear. Here, we neither could detect spontaneous induction of apoptosis nor modulation of MG‑132- and Bax-induced apoptosis upon overexpression of sCLU/CLU_1‑449_, CLU_21‑449_ or CLU_34‑449_. Thus we cannot confirm previous studies showing both, anti-apoptotic [41,42] as well as pro-apoptotic [38,43] functions of CLU. We suggest that the reported effects of sCLU and cytosolic CLU isoforms on intrinsic apoptosis may either depend on a specific cellular context or represent responses that are restricted to certain cell types or cell lines. Interestingly, similar possibilities are being discussed regarding the role of cytosolic PrP on cell viability [69].

Luciferase reporter assays revealed a significant inhibitory effect of CLU_34‑449_ on NF-κB -activity only when overexpressed from variant 1 [Δex2] but not from point-mutated variant 1 cDNA. Since the amount of CLU_34‑449_ translated from variant 1 [Δex2] vastly exceeds that translated from the point-mutated variant 1 cDNA indicates that this isoform influences NF-κB-activity in a dose-dependent manner. However, a physiological relevance of this effect seems unlikely, as respective amounts of CLU_34‑449_ are not reached endogenously, even under conditions of massive stress.

Regardless of the complexity of CLU proteins and functions and their still incompletely understood influence on cell viability and apoptosis, a current strategy to optimize the treatment of androgen-independent prostate cancer is to minimize sCLU synthesis while leaving cytosolic CLU expression unaffected in order to make cancer cells more susceptible to chemotherapeutic drugs. However, on the basis of our results, it may be difficult to accomplish by common antisense oligonucleotide or RNAi strategies. The approach of Essabani and colleagues to force exon 2 skipping of CLU mRNAs by suppressing an exon splicing enhancer using hairpin oligonucleotides seems more promising. This leads to a decrease in sCLU expression and a concomitant increase in the synthesis of CLU_34‑449_ resulting in a higher mortality of LNCaP prostate cancer cells [70]. On the basis of our results it is reasonable that this strategy would suppress the expression of sCLU, but also CLU_1‑449_ and CLU_21‑449_, while increasing CLU_34‑449_. However, our data indicate that none of the rare cytosolic CLU isoforms reduces cell viability. Therefore, in contrast to sCLU, these isoforms appear likely to be irrelevant in the context of cancer and other pathologies.

## Supporting Information

Figure S1 **BC010514.1 is the predominantly expressed CLU mRNA variant 1 and can be spliced to produce variant 1 [Δex2].** (A) Different entries for CLU mRNA variant 1 exist in the NCBI database having various lengths at their 5’ ends. 5’ RACE-PCR analyses of 6 different cell lines produces a DNA fragment (upper panel) that is identical to the EST BP211675 and highly similar to the 5’ end of mRNA BC010514.1 but not to that of NM_001831.3 or NM_001831.1 (lower panel). Hence, the canonical transcription start site of CLU mRNA variant 1 is located 23 nucleotides downstream of the TATA promotor element, as expected. (B) Semi-quantitative RT‑PCR analyses of CLU variant 1 mRNA expression in unstressed HEK‑293, PC‑3, MCF‑7 and Caco-2 cells using primer sets specific for BC010514.1 or NM_001831.3. Upon using 22 cycles of PCR-amplification the expression of BC010514.1 is observed in all cell lines. CLU mRNA NM_001831.3, however, is expressed in minor amounts only in HEK‑293 cells. (C) Semi-quantitative RT‑PCR analyses of CLU mRNA NM_001831.3 expression in unstressed and MG‑132 treated HEK‑293, PC‑3, MCF‑7 and Caco-2 cells using 35 cycles of PCR-amplification. CLU mRNA NM_001831.3 shows low abundant expression in HEK‑293, MCF‑7 and Caco-2 cells and a cell line specific pattern of regulation upon MG‑132 treatment. (D) RT‑PCR analysis of CLU variant 1 mRNA and variant 1 [Δex2] mRNA expression in MG‑132 treated PC‑3 cells using variant 1-specific primers and 35 cycles of amplification. Specificity of both resulting PCR products was verified by DNA sequencing. They represent variant 1 mRNA containing exon 2 (+ exon 2) and variant 1 [Δex2] (Δ exon 2). (E) Plasmids carrying variant 1 or variant 1 [Δex2] cDNA served as templates for PCRs performed with a variant 1- (upper panel) or a variant 1 [Δex2]-specific primer set (lower panel). While both cDNAs can be detected by variant 1-specific primers resulting in the amplification of two PCRs with different length, variant 1 [Δex2]-specific primers solely detect variant 1 [Δex2] cDNA. When mixtures of both cDNAs with ratios of variant 1: variant 1 [Δex2] = 100:1 or higher were used as a template, variant 1 [Δex2] cDNA is only detectable by variant 1 [Δex2] primers, but no longer by the variant 1 primer set. Considering that in cells usually amounts of variant 1mRNA exceed those of variant 1 [Δex2] by four orders of magnitude does result in difficulties when detecting the CLU variant 1 [Δex2] mRNA by conventional variant 1 specific primer sets.
(PDF)

Figure S2 **Upstream ORFs impair sCLU translation from variants 2 and 3 whereas an upstream in-frame start codon on NM_001831.3 initiates translation of sCLU.** (A+B) Western blot analysis of whole cell lysates (50 µg total protein) and cell culture media (30 µl) of HEK‑293 cells transiently expressing unmodified or point-mutated versions of the indicated CLU cDNA variants. Recombinant CLU protein was detected using the V5-tag specific antibody. Cells transfected with blank pcDNA6 (mock) served as controls (lanes 1). Analysis of α-tubulin was performed as a loading control. Lanes are labeled with circled numbers. Data shown are representative of three independent experiments. (A) Schematic outlines of the 5’-ends of cDNA variants 2 and 3 are shown. Exon 1 sequences of both variants contain a set of uORFs (indicated by brackets) which differ from the CLU reading frame. On each variant the longest uORF (* or **) overlaps with the CLU reading frame leading to lower expression of sCLU compared to variant 1, which does not contain any uORFs (lanes 2, 3, 5). Point-mutation of the start codons of these uORFs leads to an increase in the amount of sCLU expressed from variant 2 and 3 which is comparable to that synthesized from variant 1 (lanes 4, 6), strongly indicating that these uORFs inhibit translation initiation at the sCLU start codon (framed) as well as the alternative sCLU start codon on variant 3. (B) A schematic outline of the 5’-end of the 5’-extended cDNA variant 1 (NM_001831.3) is shown. Neither point-mutations of the sCLU start codon (framed) nor the in-frame ATG on exon 1, which is part of the 5’-extended exon 1a sequence (dark grey box), do inhibit sCLU expression, indicating that both codons initiate sCLU translation. Concurrent mutation of both codons, however, almost completely blocks sCLU synthesis. Note that ATG on exon 1a also initiates the translation of a 60 kDa CLU protein that likely represents an N-terminal elongated sCLU pre-proprotein corresponding to CLU_1‑449_ expressed from variant 1 (BC010514.1). Respective mutated start sites of modified cDNAs are indicated above each lane (crossed out).
(PDF)

Figure S3 **Subcellular localization of individual CLU isoforms.** HEK‑293 cells were transfected with unmodified variant 1, variant 1 [Δex2] or point-mutated versions of variant 1 cDNA encoding only sCLU/CLU_1‑449_, CLU_21‑449_ or CLU_34‑449_ and subjected to LSM. CLU-V5 was detected by the anti‑V5 primary antibody and the Cy3-conjugated secondary antibody (red). Alexa Fluor^®^ 488-conjugated WGA (green) and DAPI (blue) served as counterstains for Golgi/plasmamembrane and the nucleus, respectively. Images shown represent the middle plane of the analyzed cells. When unmodified variant 1 cDNA or sCLU/CLU_1‑449_ are expressed the staining of CLU and WGA shows an overlay (yellow) caused by the presence of psCLU in the ER (variant 1, sCLU/CLU_1‑449_, control). Expression of variant 1 [Δex2] leads to a mutual exclusive CLU and WGA staining (variant 1 [Δex2], control). A similar staining is observed for CLU_21‑449_ and CLU_34‑449_ (CLU_21‑449_, CLU_34‑449_, control). The presence of 10 µM MG‑132 does not lead to alterations in the subcellular localization of the individual CLU isoforms when compared to untreated controls. The disruption of intracellular membranes, condensed chromatin and nuclear fragmentation is indicative for apoptotic processes induced by MG‑132 treatment.
(PDF)

Figure S4 **Impact of individual CLU isoforms on apoptosis of PC-3 cells.** PC-3 cells were transfected with pcDNA6 (mock), unmodified variant 1 (wildtype), variant 1 [Δex2] (Δex2) or point-mutated versions of variant 1 cDNA encoding only sCLU/CLU_1‑449_, CLU_21‑449_ or CLU_34‑449_. 24 hours after transfection the activity of caspases 3 and 7 was determined. Data are expressed as fold changes in caspase activity compared to mock-transfected cells (mean ± SD, n = 3). In contrast to Bax-overexpression, which served as positive control, the expression of all CLU protein forms does not activate caspase‑3/7.
(PDF)

Protocol S1 **Trial protocol.**
(DOCX)

Table S1 **Sequences of DNA oligomers which were used as primers for semi-quantitative RT PCR, quantitative real-time PCR and 5’ RACE.**
(DOCX)

Video S1 **(A) Animated LSM Z stack of MG 132-treated HEK 293 cells expressing sCLU/CLU_1-449_.** CLU-V5 was detected by the anti V5 primary antibody and the Cy3-conjugated secondary antibody (red). Alexa Fluor® 488-conjugated ConA (green) and DAPI (blue) served as counterstains for the nuclear membrane-ER continuum and the nucleus, respectively. Staining of CLU and ConA shows an overlay (yellow). (B) Animated LSM Z stack of MG 132-treated HEK 293 cells expressing CLU_21-449_. CLU-V5 was detected by the anti V5 primary antibody and the Cy3-conjugated secondary antibody (red). Alexa Fluor® 488-conjugated ConA (green) and DAPI (blue) served as counterstains for the nuclear membrane-ER continuum and the nucleus, respectively. (C) Animated LSM Z stack of MG 132-treated HEK 293 cells expressing CLU_34-449_ from variant 1 [Δex2] cDNA. CLU-V5 was detected by the anti V5 primary antibody and the Cy3-conjugated secondary antibody (red). Alexa Fluor® 488-conjugated WGA (green) and DAPI (blue) served as counterstains for the Golgi/plasma membrane and the nucleus, respectively.
(ZIP)
