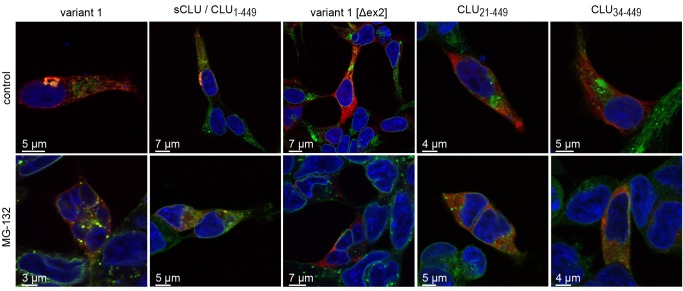# Correction: Non-Secreted Clusterin Isoforms Are Translated in Rare Amounts from Distinct Human mRNA Variants and Do Not Affect Bax-Mediated Apoptosis or the NF-κB Signaling Pathway

**DOI:** 10.1371/annotation/48cea21c-d035-4757-bf42-c97028306f11

**Published:** 2013-11-12

**Authors:** Hans Prochnow, Rene Gollan, Philipp Rohne, Matthias Hassemer, Claudia Koch-Brandt, Markus Baiersdörfer

The quality of the version of Figure 6 that appears in the article does not allow for the reproduction of certain data.

A correct version of the figure is available here: 

**Figure:**