# Supplementary figures and images for: Non-Secreted Clusterin Isoforms Are Translated in Rare Amounts from Distinct Human mRNA Variants and Do Not Affect Bax-Mediated Apoptosis or the NF-κB Signaling Pathway
Source: PLoS One. 2013 Sep 20;8(9):e75303. doi: 10.1371/journal.pone.0075303 (PMC3779157; doi:10.1371/journal.pone.0075303)

**A**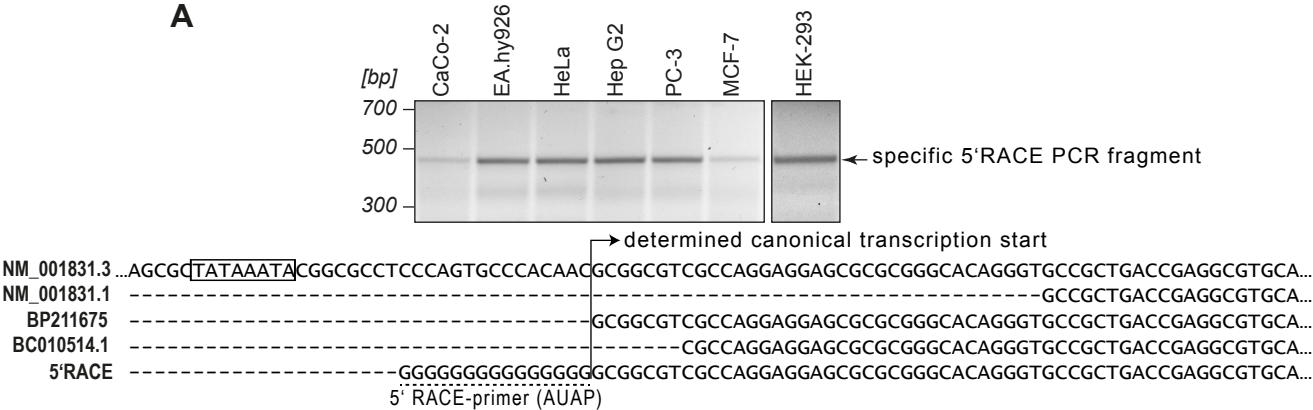**B**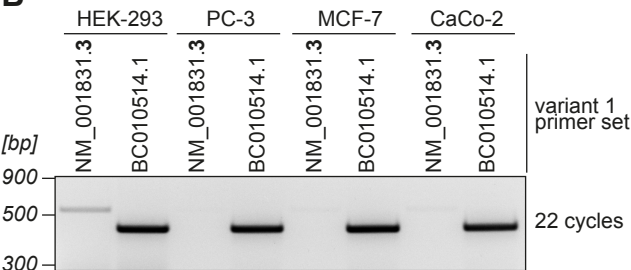**D**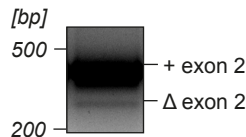**C**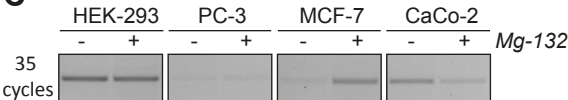**E**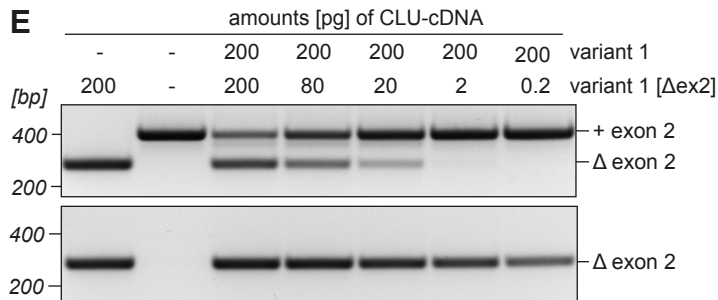

Supplement: Figure S1 — BC010514.1 is the predominantly expressed CLU mRNA variant 1 and can be spliced to produce variant 1 [Δex2]. (A) Different entries for CLU mRNA variant 1 exist in the NCBI database having various lengths at their 5’ ends. 5’ RACE-PCR analyses of 6 different cell lines produces a DNA fragment (upper panel) that is identical to the EST BP211675 and highly similar to the 5’ end of mRNA BC010514.1 but not to that of NM_001831.3 or NM_001831.1 (lower panel). Hence, the canonical transcription start site of CLU mRNA variant 1 is located 23 nucleotides downstream of the TATA promotor element, as expected. (B) Semi-quantitative RT‑PCR analyses of CLU variant 1 mRNA expression in unstressed HEK‑293, PC‑3, MCF‑7 and Caco-2 cells using primer sets specific for BC010514.1 or NM_001831.3. Upon using 22 cycles of PCR-amplification the expression of BC010514.1 is observed in all cell lines. CLU mRNA NM_001831.3, however, is expressed in minor amounts only in HEK‑293 cells. (C) Semi-quantitative RT‑PCR analyses of CLU mRNA NM_001831.3 expression in unstressed and MG‑132 treated HEK‑293, PC‑3, MCF‑7 and Caco-2 cells using 35 cycles of PCR-amplification. CLU mRNA NM_001831.3 shows low abundant expression in HEK‑293, MCF‑7 and Caco-2 cells and a cell line specific pattern of regulation upon MG‑132 treatment. (D) RT‑PCR analysis of CLU variant 1 mRNA and variant 1 [Δex2] mRNA expression in MG‑132 treated PC‑3 cells using variant 1-specific primers and 35 cycles of amplification. Specificity of both resulting PCR products was verified by DNA sequencing. They represent variant 1 mRNA containing exon 2 (+ exon 2) and variant 1 [Δex2] (Δ exon 2). (E) Plasmids carrying variant 1 or variant 1 [Δex2] cDNA served as templates for PCRs performed with a variant 1- (upper panel) or a variant 1 [Δex2]-specific primer set (lower panel). While both cDNAs can be detected by variant 1-specific primers resulting in the amplification of two PCRs with different length, variant 1 [Δex2]-specific primers s [file pone.0075303.s001.pdf]

**A**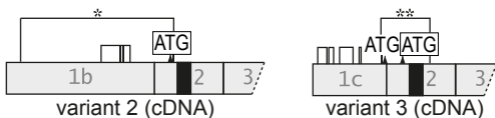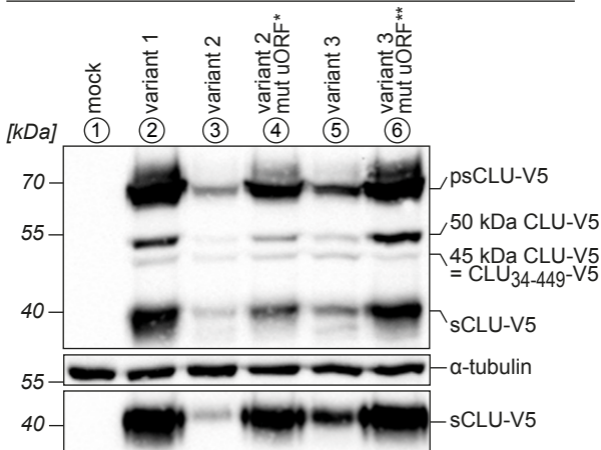**B**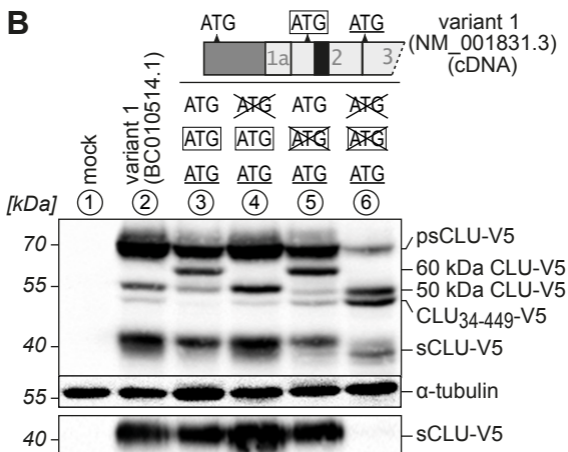

Supplement: Figure S2 — Upstream ORFs impair sCLU translation from variants 2 and 3 whereas an upstream in-frame start codon on NM_001831.3 initiates translation of sCLU. (A+B) Western blot analysis of whole cell lysates (50 µg total protein) and cell culture media (30 µl) of HEK‑293 cells transiently expressing unmodified or point-mutated versions of the indicated CLU cDNA variants. Recombinant CLU protein was detected using the V5-tag specific antibody. Cells transfected with blank pcDNA6 (mock) served as controls (lanes 1). Analysis of α-tubulin was performed as a loading control. Lanes are labeled with circled numbers. Data shown are representative of three independent experiments. (A) Schematic outlines of the 5’-ends of cDNA variants 2 and 3 are shown. Exon 1 sequences of both variants contain a set of uORFs (indicated by brackets) which differ from the CLU reading frame. On each variant the longest uORF (* or **) overlaps with the CLU reading frame leading to lower expression of sCLU compared to variant 1, which does not contain any uORFs (lanes 2, 3, 5). Point-mutation of the start codons of these uORFs leads to an increase in the amount of sCLU expressed from variant 2 and 3 which is comparable to that synthesized from variant 1 (lanes 4, 6), strongly indicating that these uORFs inhibit translation initiation at the sCLU start codon (framed) as well as the alternative sCLU start codon on variant 3. (B) A schematic outline of the 5’-end of the 5’-extended cDNA variant 1 (NM_001831.3) is shown. Neither point-mutations of the sCLU start codon (framed) nor the in-frame ATG on exon 1, which is part of the 5’-extended exon 1a sequence (dark grey box), do inhibit sCLU expression, indicating that both codons initiate sCLU translation. Concurrent mutation of both codons, however, almost completely blocks sCLU synthesis. Note that ATG on exon 1a also initiates the translation of a 60 kDa CLU protein that likely represents an N-terminal elongated sCLU pre-proprotein corresponding to CLU1‑449 [file pone.0075303.s002.pdf]

control

variant 1

sCLU / CLU<sub>1-449</sub>variant 1 [ $\Delta$ ex2]CLU<sub>21-449</sub>CLU<sub>34-449</sub>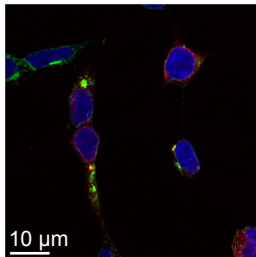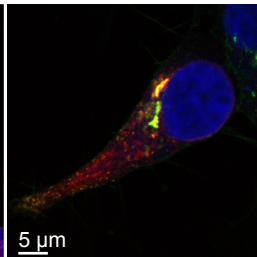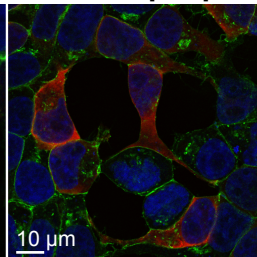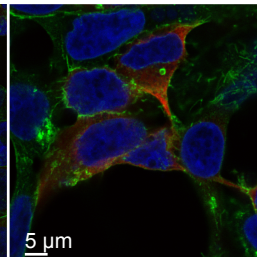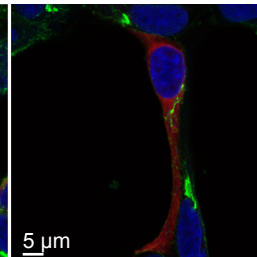

MG-132

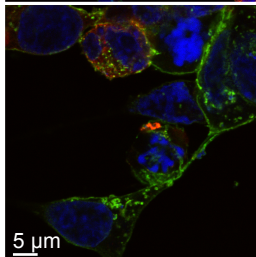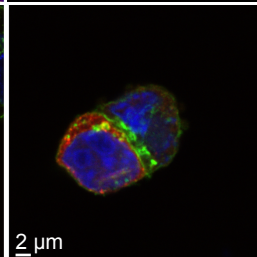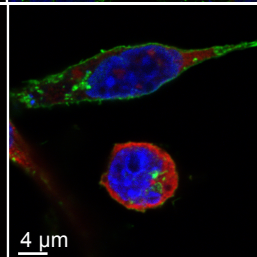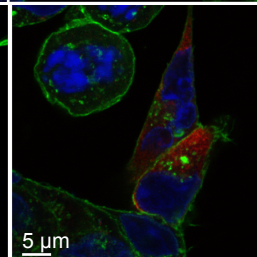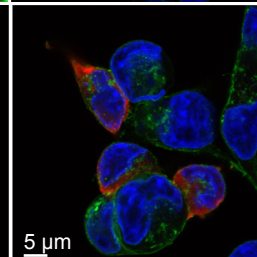

Supplement: Figure S3 — Subcellular localization of individual CLU isoforms. HEK‑293 cells were transfected with unmodified variant 1, variant 1 [Δex2] or point-mutated versions of variant 1 cDNA encoding only sCLU/CLU1‑449, CLU21‑449 or CLU34‑449 and subjected to LSM. CLU-V5 was detected by the anti‑V5 primary antibody and the Cy3-conjugated secondary antibody (red). Alexa Fluor® 488-conjugated WGA (green) and DAPI (blue) served as counterstains for Golgi/plasmamembrane and the nucleus, respectively. Images shown represent the middle plane of the analyzed cells. When unmodified variant 1 cDNA or sCLU/CLU1‑449 are expressed the staining of CLU and WGA shows an overlay (yellow) caused by the presence of psCLU in the ER (variant 1, sCLU/CLU1‑449, control). Expression of variant 1 [Δex2] leads to a mutual exclusive CLU and WGA staining (variant 1 [Δex2], control). A similar staining is observed for CLU21‑449 and CLU34‑449 (CLU21‑449, CLU34‑449, control). The presence of 10 µM MG‑132 does not lead to alterations in the subcellular localization of the individual CLU isoforms when compared to untreated controls. The disruption of intracellular membranes, condensed chromatin and nuclear fragmentation is indicative for apoptotic processes induced by MG‑132 treatment. (PDF) [file pone.0075303.s003.pdf]

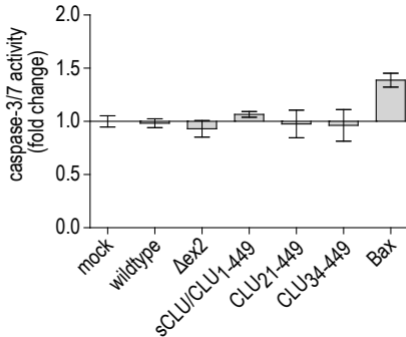

Supplement: Figure S4 — Impact of individual CLU isoforms on apoptosis of PC-3 cells. PC-3 cells were transfected with pcDNA6 (mock), unmodified variant 1 (wildtype), variant 1 [Δex2] (Δex2) or point-mutated versions of variant 1 cDNA encoding only sCLU/CLU1‑449, CLU21‑449 or CLU34‑449. 24 hours after transfection the activity of caspases 3 and 7 was determined. Data are expressed as fold changes in caspase activity compared to mock-transfected cells (mean ± SD, n = 3). In contrast to Bax-overexpression, which served as positive control, the expression of all CLU protein forms does not activate caspase‑3/7. (PDF) [file pone.0075303.s004.pdf]
