# Supplementary material for: Non-Secreted Clusterin Isoforms Are Translated in Rare Amounts from Distinct Human mRNA Variants and Do Not Affect Bax-Mediated Apoptosis or the NF-κB Signaling Pathway
Source: PLoS One. 2013 Sep 20;8(9):e75303. doi: 10.1371/journal.pone.0075303 (PMC3779157; doi:10.1371/journal.pone.0075303)
Supplement: Protocol S1 — Trial protocol. (DOCX) [file pone.0075303.s005.docx]

# Protocol S1. Materials and methods

## Cell culture

Human embryonic kidney cells (HEK-293) were a gift from Dr. R. Postina (University of Mainz, Germany) and maintained in Dulbecco’s Modified Eagle’s medium (DMEM) supplemented with 1 mM sodium pyruvate, 2 mM L-glutamine and 10% FBS. Androgen-independent, metastatic human prostate cancer cells (PC-3) were a gift from Dr. S. Bettuzzi (University of Parma, Italy). PC-3 cells were grown in DMEM containing 2 mM L-glutamine and 10% FBS. Human mammary gland carcinoma cells (MCF-7) were obtained from Dr. K. Endres (University of Mainz, Germany) and cultivated in phenol-red containing RPMI 1640 medium supplemented with 2 mM L-glutamine and 10% FBS. The human colorectal adenocarcinoma cell line Caco-2 was obtained from Dr. P. Langguth (University of Mainz, Germany) and maintained in MEM-Earle’s medium containing 1 mM sodium pyruvate, 2 mM L-glutamine, non-essential amino acids and 10% FBS. Cell culture reagents were purchased from Biochrom.

## Preparation of cell lysates and Western blot analysis

First of all culture medium was removed and centrifuged at 500 × g to remove floating cells. Cells were lysed in ice-cold lysis buffer (50 mM Tris/HCl [pH 8], 150 mM NaCl, 1% (v/v) Triton^®^ X‑100) containing protease inhibitor (Complete mini, Roche) and incubated on a rotary shaker for 1 h followed by 30 minutes of centrifugation at 20,000 × g to obtain cleared cell lysates. Protein concentrations were determined by the Bradford assay using Roti^®^‑Quant (Roth). Deglycosylation of proteins was carried out by digestion of 40 µg total protein with 1,000 units PNGase F (NEB) according to the manufacturer’s protocol. For Western blot analysis 40-150 µg of total protein or 30-40 µl of culture medium were subjected to reducing SDS‑PAGE and blotted onto nitrocellulose membranes. The polyclonal antibody sc‑6419 (1:1,000 dilution, Santa Cruz) was used for detection of untagged human CLU and monoclonal anti‑V5 antibody (1:5,000 dilution, Life Technologies) for detection of tagged CLU. Human α‑Tubulin was analysed via a monoclonal antibody (1:5,000 dilution, Sigma). Peroxidase conjugated secondary antibodies (1:10,000 dilution) and Western Lightning^®^‑ECL substrate (Perkin Elmer) were used to visualize reactive bands. Chemiluminescence reactions were imaged using the VersaDoc™ system (Bio Rad). Coomassie staining of SDS‑PAGE gels was performed with Roti^®^‑Blue (Roth).

## Reverse transcription, real-time PCR and 5’ RACE analyses

Total cellular RNA was isolated using the innuPrep RNA Mini Kit (Analytic Jena) according to the manufacturer’s protocol. 5 µg total RNA were applied for reverse transcription with oligo dT primer (Roth) and RevertAid™ reverse transcriptase (Thermo Scientific). All PCRs were performed using 20 ng/µl of reverse transcribed RNA at an annealing temperature of 60°C. For semi-quantitative PCR analyses, the Maxima™ Hot Start Green PCR Master Mix (Thermo Scientific) was used. Quantitative real-time PCR analyses were performed with the SYBR green / ROX based RealQ PCR Master Mix (Biomol). Quantification of mRNAs was performed in triplicates using the 7500 Fast System and SDS Software (Applied Biosystems). Plasmids carrying the respective CLU cDNAs at concentrations ranging from 10^3^ - 10^‑5^ pg/µl served as standards for the calculation of mRNA copy numbers per ng of total RNA. All PCR fragments amplified were verified by DNA sequencing.

## Immunocytochemistry

HEK‑293 cells were grown on coverslips (Ø 1 cm) and transfected with recombinant CLU cDNAs. If indicated, the cells were treated with 10 µM MG‑132 as described above. The cells were fixed with 4% (w/v) paraformaldehyde and quenched with PBS containing 50 mM ammonium chloride. Following permeabilization with PBS/0.05% (v/v) Triton^®^ X‑100 cells were incubated with Alexa Fluor^®^ 488 conjugated lectins Concanavalin A (ConA) or wheat germ agglutinin (WGA) at concentrations of 50 µg/ml and 10 µg/ml, respectively (Life Technologies). ConA interacts with a broad spectrum of N‑glycosylated proteins, especially with high mannose glycoproteins in the ER compartment while WGA binds to terminal N‑acetylglucosamine and sialic acid residues, found in glycoproteins with complex N- and O‑glycans present in the trans-Golgi and the cell membrane [1]. After blocking with PBS/2% (w/v) BSA cells were incubated consecutively with anti‑V5 antibody (1:5,000 dilution, Life Sciences) and Cy3-conjugated secondary antibody (1:250 dilution, Dianova) followed by chromatin staining with 0,2 µg/ml DAPI in PBS. Cells were imaged by confocal LSM at a Z‑stack step size of 0.13 µm with a 63× oil immersion objective (1.4 optical aperture) using the LSM SP5 microscope (Leica) and Imaris software (Bitplane).

## Determination of caspase‑3/7 activity

1.5 × 10^4^ HEK‑293 cells were cultivated in 96‑wells and transfected with 0.2 µg of plasmid DNA. After 18 hours cells were treated for 10 hours with 10 µM MG‑132 or an equivalent volume of DMSO. Caspase activity was determined using the Caspase‑Glo^®^‑3/7 Assay (Promega) according to the manufacturer’s protocol and FLUOstar Omega luminometer with Omega‑Data Analysis Mars software (BMG Labtech).

## Literature

1. Sparbier K, Wenzel T, Kostrzewa M (2006) Exploring the binding profiles of ConA, boronic acid and WGA by MALDI-TOF/TOF MS and magnetic particles. J Chromatogr B Analyt Technol Biomed Life Sci 840 (1):29-36. doi:S1570-0232(06)00526-5 [pii]

10.1016/j.jchromb.2006.06.028
