# Supplementary material for: Non-Secreted Clusterin Isoforms Are Translated in Rare Amounts from Distinct Human mRNA Variants and Do Not Affect Bax-Mediated Apoptosis or the NF-κB Signaling Pathway
Source: PLoS One. 2013 Sep 20;8(9):e75303. doi: 10.1371/journal.pone.0075303 (PMC3779157; doi:10.1371/journal.pone.0075303)
Supplement: Table S1 — Sequences of DNA oligomers which were used as primers for semi-quantitative RT PCR, quantitative real-time PCR and 5’ RACE. (DOCX) [file pone.0075303.s006.docx]

Table S1: Sequences of DNA oligomers which were used as primers for semi-quantitative RT‑PCR, quantitative real-time PCR and 5’ RACE

| **quantitative PCR primers** | **forward (location)** | **reverse (location)** | **amplicon size** |
| --- | --- | --- | --- |
| total CLU | CTATCTGCGGGTCACCAC (exon 7) | CTCAGTGACACCGGAAGGAAC (exon 8) | 71 bp |
| CLU variant 1 | ACAGGGTGCCGCTGACC (exon 1a) | CAGCAGAGTCTTCATCATGCC (exon 2) | 65 bp |
| CLU variant 2 | ATGCAGATGGATTCGGTGT (exon 1b) | AGTCTTTGCACGCCTCTGA (exon 2) | 80 bp |
| CLU variant 3 | TCGTCCTGTTGGTTCTGTGATG (exon 1c) | CAGCAGAGTCTTCATCATGCC (exon 2) | 77 bp |
| CLU variant 1 Δ exon 2 | GGGTGCCGCTGACCGAAAT (exon 1a / exon 3) | GAGTCTTTATCTGTTTCACCCCG (exon 3) | 93 bp |
|  |  |  |  |
| **semi-quantitative PCR primers** |  |  |  |
| hsp27 | GGAGTGGTCGCAGTGGTTAGG | GGGAGGAGGAAACTTGGGTG | 350 bp |
| gapdh | GCCAAAAGGGTCATCATCTC | GCTTCACCACCTTCTTGATGTC | 445 bp |
| total CLU | AAAATGCTGTCAACGGGGTG (exon 3) | TTCAGGCAGGGCTTACACTCT (exon 5) | 214 bp |
| CLU variant 1 | ACAGGGTGCCGCTGACC (exon 1a) | TTCAGGCAGGGCTTACACTCT (exon 5) | 400 bp |
| CLU variant 2 | CACTGCGAACCCTCTCTACTCTC (exon 1b) | TTCAGGCAGGGCTTACACTCT (exon 5) | 550 bp |
| CLU variant 3 | TCGTCCTGTTGGTTCTGTGATG (exon 1c) | TTCAGGCAGGGCTTACACTCT (exon 5) | 412 bp |
| CLU variant 1 Δ exon 2 | GGGTGCCGCTGACCGAAAT (exon 1a / exon 3) | TTCAGGCAGGGCTTACACTCT (exon 5) | 271 bp |
| CLU variant 3 Δ exon 2 | TTCTGTGATGGCAAATGGAAAT (exon 1c / exon 3) | TTCAGGCAGGGCTTACACTCT (exon 5) | 274 bp |
| CLU variant 1 [ext] | CTTTCCGCGGCATTCTTTG (exon 1a) | TTCAGGCAGGGCTTACACTCT (exon 5) | 538 bp |
|  |  |  |  |
| **5'RACE primers** |  |  |  |
| Gene specific primer (GSP) 1 | - | CTCAGTGACACCGGAAGGAAC (exon 8) |  |
| GSP 2 | - | GTGCGTAGAACTTCATGCA (exon 5) |  |
| GSP 3 | - | TTCAGGCAGGGCTTACACTCT (exon 5) |  |
| Abridged anchor primer (AAP) | GGCCACGCGTCGACTAGTACGGGIIGGGIIGGGIIG | - |  |
| Abridged universal amplification primer (AUAP) | GGCCACGCGTCGACTAGTAC | - |  |
